# An efficient algorithm for identifying primary phenotype attractors of a large-scale Boolean network

**DOI:** 10.1186/s12918-016-0338-4

**Published:** 2016-10-07

**Authors:** Sang-Mok Choo, Kwang-Hyun Cho

**Affiliations:** 1 Department of Mathematics, University of Ulsan, Ulsan, 44610 Republic of Korea; 2 Department of Bio and Brain Engineering, Korea Advanced Institute of Science and Technology (KAIST), Daejeon, 34141 Republic of Korea

**Keywords:** Boolean network, Cell phenotypes, Attractors, Hierarchical partition, Systems biology

## Abstract

**Background:**

Boolean network modeling has been widely used to model large-scale biomolecular regulatory networks as it can describe the essential dynamical characteristics of complicated networks in a relatively simple way. When we analyze such Boolean network models, we often need to find out attractor states to investigate the converging state features that represent particular cell phenotypes. This is, however, very difficult (often impossible) for a large network due to computational complexity.

**Results:**

There have been some attempts to resolve this problem by partitioning the original network into smaller subnetworks and reconstructing the attractor states by integrating the local attractors obtained from each subnetwork. But, in many cases, the partitioned subnetworks are still too large and such an approach is no longer useful. So, we have investigated the fundamental reason underlying this problem and proposed a novel efficient way of hierarchically partitioning a given large network into smaller subnetworks by focusing on some attractors corresponding to a particular phenotype of interest instead of considering all attractors at the same time. Using the definition of attractors, we can have a simplified update rule with fixed state values for some nodes. The resulting subnetworks were small enough to find out the corresponding local attractors which can be integrated for reconstruction of the global attractor states of the original large network.

**Conclusions:**

The proposed approach can substantially extend the current limit of Boolean network modeling for converging state analysis of biological networks.

**Electronic supplementary material:**

The online version of this article (doi:10.1186/s12918-016-0338-4) contains supplementary material, which is available to authorized users.

## Background

In the realm of systems biology, mathematical modeling is essential to unravel the hidden principles underlying complex biological phenomena [1]. Among various mathematical modeling frameworks, the Boolean network is particularly useful for modeling large-scale biomolecular regulatory networks as it is a parameter-free logical model and thereby we can avoid parameter estimation which is often a critical limitation in mathematical modeling of such large-scale networks [2–5]. Once a Boolean network model is obtained, the converging state characteristics of the modeled network can be investigated by identifying attractor states which were known corresponding to cell phenotypes [6–11]. Finding attractors of interest is, however, an NP-hard problem [12–14] since we have to search the full state space and this is only possible for small networks with less than about 20 nodes [15, 16].

To tackle such a problem, there have been several attempts to reduce the original Boolean network model by eliminating some nodes or logically simplifying Boolean functions [17–27], or focusing only on point attractors [28, 29]. Another attempt was partitioning the original large Boolean network into smaller blocks and reconstructing the original attractors by integrating the local attractors of partitioned blocks [30, 31]. However, none of these could resolve the fundamental problem of computational complexity since both the reduced network and the partitioned subnetwork are still too large in most cases of biological networks. Even if the reduced network is small enough to search the full state space, the resulting attractor states of the reduced network can be different from the attractor states of the original network (this will be shown in the Results section). The existing partitioning approaches retain the complicated logic of the original network even after partitioning such that the whole set of attractors can still be found from the partitioned subnetworks. We found that the fundamental limitation of the previous partitioning methods lies in this point. To overcome such a limitation, we propose a different approach in this paper. The main idea is focusing only on particular phenotypic attractors of interest and simplifying the Boolean update rules of the original network by introducing some constraint equations such that the particular attractors are not affected. In this way, we can efficiently reduce the original large network. Then, we further partition the reduced network hierarchically and find out the local attractors of each partitioned subnetwork. We can finally obtain the global attractors for representing the particular phenotype in the original network by sequentially concatenating the local attractors. Our approach is based on the previous concept of strongly connected component (SCC) [30, 31], but the main difference is that our approach can efficiently find out the particular phenotypic attractors of interest by hierarchically partitioning the reduced network obtained by simplifying the state update rules using some constraint equations, whereas the previous approach attempts to partition the original network while retaining all the complicated state update rules and thereby results in still a large subnetwork even after partitioning.

We validated the usefulness of our approach by applying it to several large and complicated biological Boolean network models.

## Methods

In this section, we describe the procedure of finding global attractors for the phenotype of interest from a large and strongly connected network and explain how to hierarchically partition the network into smaller-size subnetworks. We then describe a way of constructing the global attractors by sequential concatenation of local attractors of the subnetworks.

### Procedure for construction of global attractors by concatenating local attractors

**Fig. 1:**
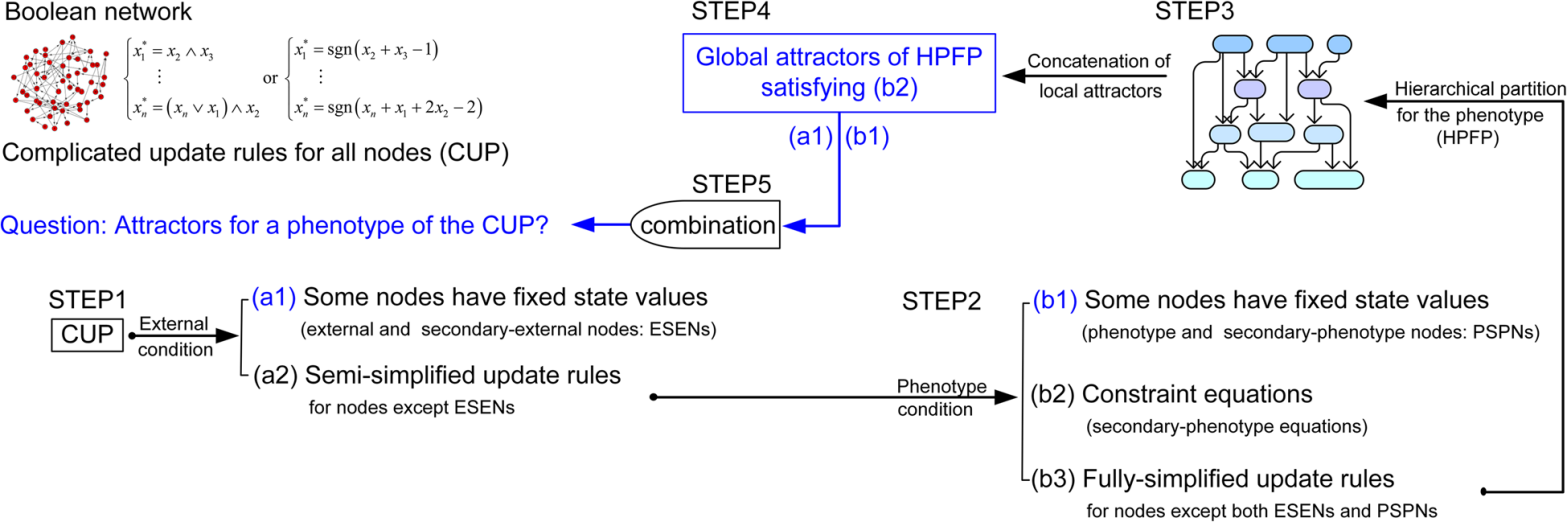
The overall procedure of the proposed method. The STEPs explain how to find the attractors for a phenotype of interest in a given Boolean network step by step. The environment of the network defines the external condition in STEP1. The consistent activation of nodes for representing the phenotype provides the phenotype condition in STEP2, where the desired global attractor states for the proliferation phenotype must satisfy the constraint equations (b2). The network with the fully-simplified update rules (b3) is partitioned in STEP3. Concatenating the local attractors obtained from each partition yields the global attractors of the fully-simplified network in STEP4. Finally, in STEP5, the global attractors of the original network can be found by combining the global attractors of the simplified network in STEP4 and the fixed state values of ESENs (a1) and PSPNs (b1)
- Step 2-1. Determine the nodes and their fixed state values for the definition of the phenotype. We consider networks with a node that is the phenotype as in [6, 9, 10]. For instance, in case that the network has the state update rule, Proliferation* = p70 & MYC & !p21, the network is said to have the node for proliferation, where the symbols * and (&,!) denote the next time step and the logic operators (and, or), respectively. Consistent activation of the phenotype is defined by both nodes and their fixed state values, which are referred to as “phenotype nodes and values”. For the update rule, Proliferation* = p70 & MYC & !p21, the phenotype nodes are (p70, MYC, p21) with fixed values (1,1,0). Therefore the desired global attractor states for the proliferation phenotype must satisfy the constraints (p70, MYC, p21) = (1,1,0). The step is described in detail in Additional file 1.
- Step 2-2. Insert the fixed values of the phenotype nodes into the semi-simplified update rules. After applying Step 2-2, we can obtain the fixed state values of some nodes, which are neither ESENs nor phenotype nodes and referred to as “secondary-phenotype nodes”. Consequently, the semi-simplified update equations are divided into three parts. The first is the set of phenotype and secondary-phenotype nodes (PSPNs) with their fixed values. The second is constraint equations, which are introduced due to the constraints in Step 2-1 and explained in S.Example 3 in Additional file 1. The third is the new update rules for nodes except both ESENs and PSPNs, which are called as “the fully-simplified update rules”. The three parts are referred to as “the phenotype condition” (Fig. 1 STEP2). Examples for Step 2 are given in Additional files 2(d), 3(c), 4(d), 5(c) and 6(d). Note that if update rules are defined by threshold (sign) functions, then constraint equations are changed to constraint inequalities as in the SCP network of the Result section and S.Remark 1 of Additional file 1.
- Step 3. Construct the hierarchical partition of the network corresponding to the fully-simplified update rules. Note that this partition is for nodes except both ESENs and PSPNs not for all nodes in the original network. The partition is referred to as “the hierarchical partition for the phenotype (HPFP)” as in Fig. 1 STEP3 and this step is explained in the Construction of HPFP section below. Examples for Step 3 are shown in Fig. 2 and the Result section.

Let us consider that a large and complicated synchronous Boolean network is given and the states of nodes in the network are updated by logic functions or threshold (sign) functions. Our goal is to find attractors for a phenotype of interest (for example, apoptosis or proliferation) in the given network. The idea is to transform the original update rules into simplified update rules by fixing the state values of some nodes (Steps 1 and 2) and to convert the original network into a simplified network using the simplified update rules. After hierarchically partitioning the simplified network into their SCCs (Step 3), the local attractors of each SCC can be found by using a full search algorithm (Step 4). Finally, we can find the global attractors by concatenating the local attractors (Step 5). Each step of the procedure is as follows:

- Step 1-1. Determine the fixed state values of nodes for external environment. The environment is a stimulus, a tumour-promoting microenvironment or perturbation of a node as in [6, 9, 10]. Nodes for representing the environment are referred to as “external nodes”.
- Step 1-2. Insert the fixed values of the external nodes into the system of update equations. Applying Step1-2, we can obtain the fixed state values of some nodes, which are different from the external nodes and referred to as “secondary-external nodes”. As a result, the original update rules are divided into two parts. The first is the set of external and secondary-external nodes (ESENs) with their fixed values. The other is the new update rules for those nodes except ESENs, which are called as “the semi-simplified update rules”. We refer to the two parts as “the external condition” (Fig. 1 STEP1). Examples for Step 1 are given in S.Example 1 in Additional file 1 (see Additional files 2(c), 3(b), 4(c), 5(b) and 6(c) for details).

**Fig. 2:**
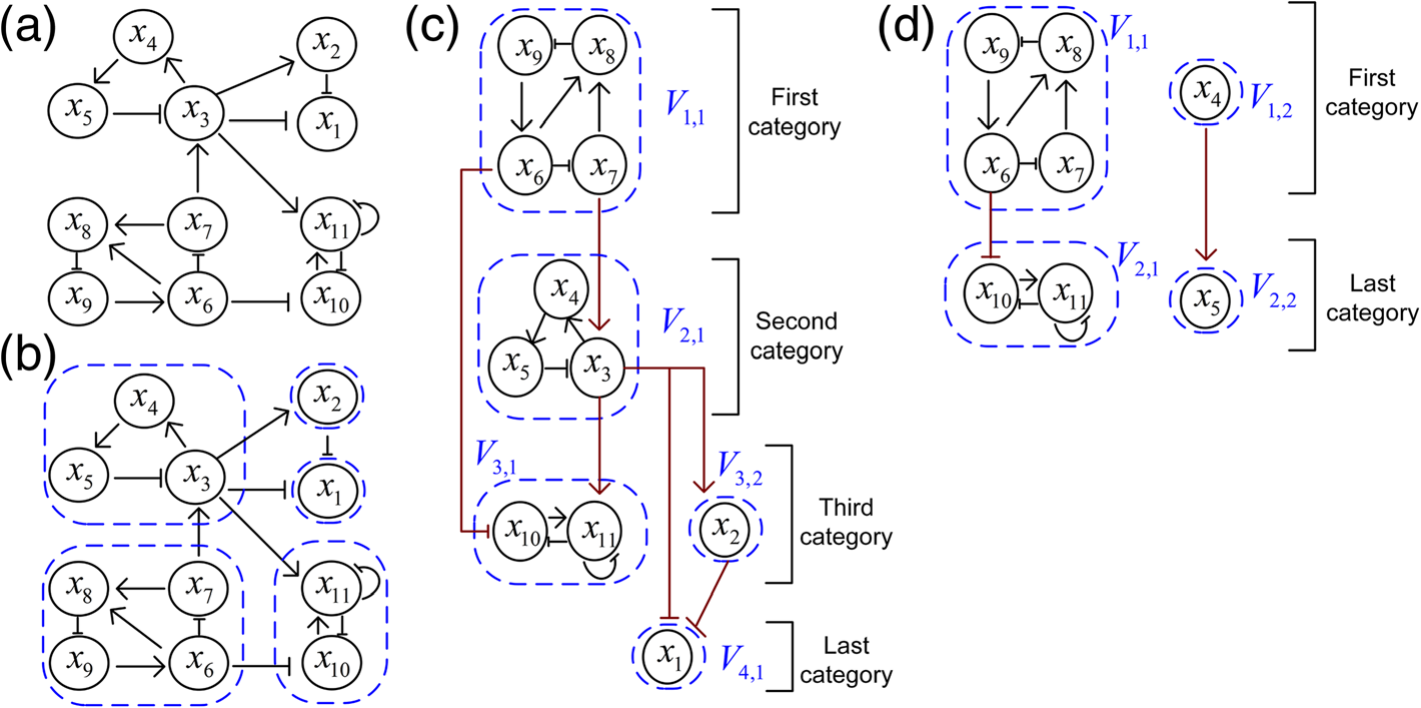
Phenotype nodes and HPFP. *Arrows* and *blunt arrows* denote activation and inhibition, respectively. *Dotted circles* means SCCs. **a**-**b** The original network has five SCCs. **c** Partition obtained from [30, 31]. **d** The HPFP is obtained under the assumption that *x*_1_ is phenotype and (*x*_2_, *x*_3_) are the phenotype nodes
- Step 4-1. Find the local attractors of subnetworks in the HPFP.
- Step 4-2. Construct the global attractors of the HPFP (Fig. 1 STEP4) by sequential concatenation of the local attractors satisfying the secondary-phenotype equations. Step 4 is explained in detail in the section of ‘How to concatenate the local attractors of subnetworks?’ and Additional files 7 and 8.
- Step 5. Combine the global attractors of the HPFP with fixed values of ESENs and PSPNs as shown in STEP5 of Fig. 1. After applying Step 5, we can obtain all the desired phenotype attractors of the original network. Examples for such phenotype attractors are given in Additional files 2(g), 3(f), 4(f)-(g), 5(f)-(m) and 6(e).

### Construction of HPFP

Let us explain how to construct the HPFP introduced in Step 3 above. The layers of the HPFP are referred to as categories. The first category consists of SCCs with zero indegree as the SCC *V*_1,1_ in Fig. 2. The n-th category (*n* ≥ 2) consists of SCCs satisfying two conditions: every link into SCCs in the n-th category comes from nodes in the k-th categories (1 ≤ *k* ≤ *n* − 1) and SCCs in the n-th category have at least one input link coming from nodes in the (n-1)-th category. The HPFP is defined as the union of the hierarchical categories, each of which is also partitioned by SCCs. The HPFP has a simpler structure than the partition of the original network as the HPFP is not a partition of the original network but of the network simplified by the fully-simplified update rules.

For instance, let us assume that there exists a Boolean network in Fig. 2a, which has five SCCs in Fig. 2b. Applying the methods in [30, 31], the Boolean network is partitioned as in Fig. 2c. The goal of the partition in [30, 31] is to make a framework for finding all attractors of the original network without fixing a particular phenotype. However, we are interested in construction of other partition (HPFP) for finding particular attractors which represent a phenotype of interest. For simplicity, we assume that there exist no external, secondary-external and secondary-phenotype nodes. In addition, we assume that *x*_1_ is a phenotype with the update equation

$$ {x}_1^{*}={f}_1\left({x}_2,{x}_3\right) $$

and that the phenotype nodes are (*x*_2_, *x*_3_) with one secondary-phenotype equation

0 = *x*_3_ = *f*_2_(*x*_5_, *x*_7_).

Hence, the nodes *x*_*i*_(1 ≤ *i* ≤ 3) and the links connected to *x*_*i*_(1 ≤ *i* ≤ 3) are removed from Fig. 2c, thus changing Fig. 2c into Fig. 2d. Therefore, the first category of the HPFP in Fig. 2d comprises *V*_1,1_ = {*x*_6_, *x*_7_, *x*_8_, *x*_9_} and *V*_1,2_ = {*x*_4_}, which have a zero indegree. The SCCs *V*_2,1_ = {*x*_10_, *x*_11_} and *V*_2,2_ = {*x*_5_} in the second category are the unique SCCs with links connected to the nodes in the first category. Comparing the two hierarchical partitions in Fig. 2c and d, we can find that the HPFP is simpler than the partition that can be obtained from previous methods [30, 31].

### How to concatenate the local attractors of subnetworks in the HPFP?

**Fig. 3:**
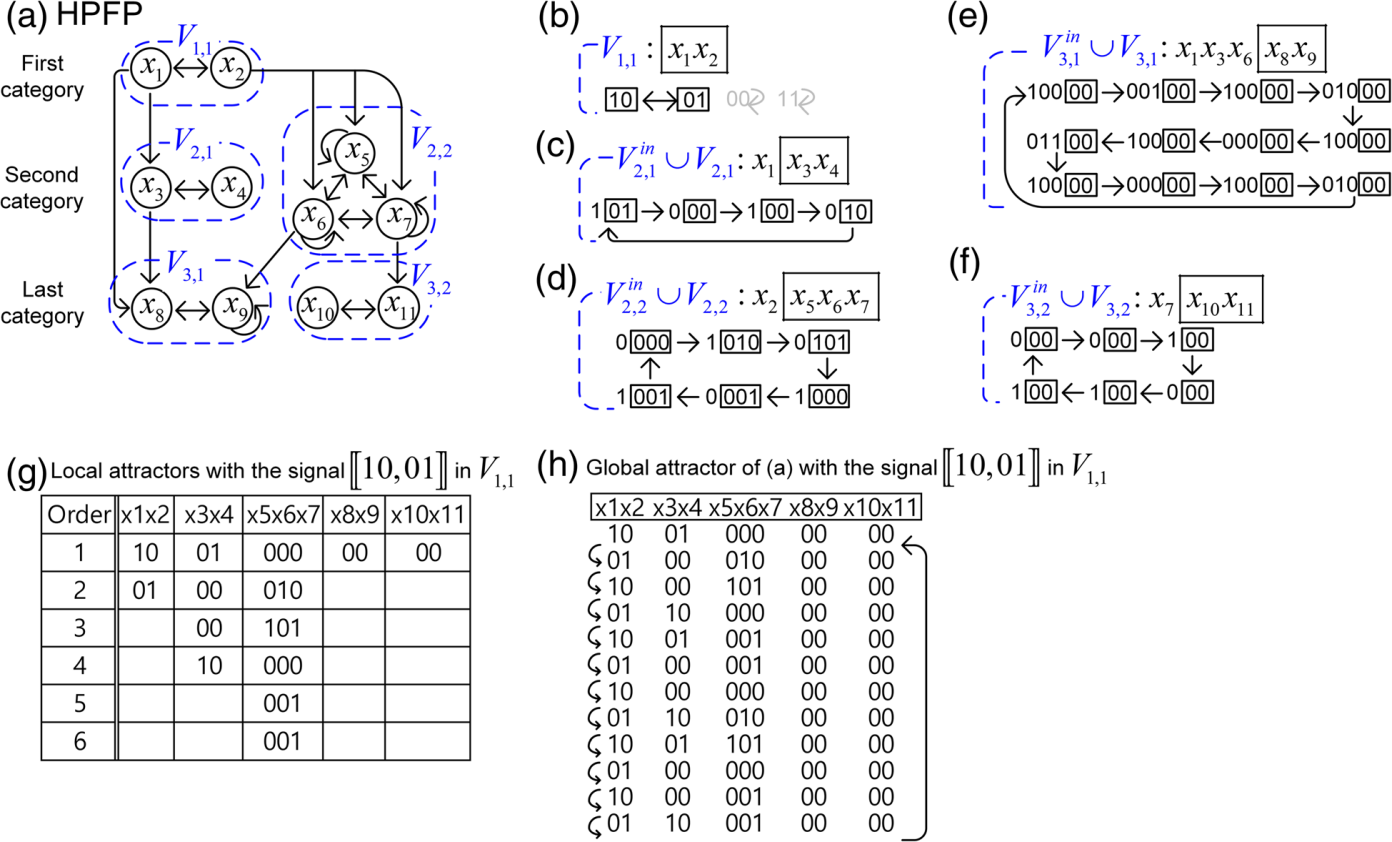
Concatenation of local attractors. **a** The HPFP has three categories and five SCCs *V*_1,1_, *V*_2,1_, *V*_2,2_, *V*_3,1_ and *V*_3,2_. Each arrow denotes the change from one state to another state at the next time step. The update rules for the network in Additional file 7. **b** There exist three attractors [10, 01], [00] and [11] in *V*_1,1_. In this figure we consider the local attractors of subnetworks in the HPFP with starting signal [10, 01] generated from the two nodes *x*_1_ and *x*_2_. **c** *V*_2,1_^*in*^ = {*x*_1_} denotes the set of nodes sending input signal into the SCC *V*_2,1_ = {*x*_3_, *x*_4_}, where the input signal is 1,0 with period 2 and *V*_2,1_ has a unique attractor [01, 00, 00, 10]. **d** *V*_2,2_^*in*^ = {*x*_2_} and *V*_2,2_ = {*x*_5_, *x*_6_, *x*_7_}. The input signal coming from *x*_2_ into *V*_2,2_ is 0, 1 with period 2. The SCC *V*_2,2_ has a unique attractor, which is cyclic with length 6. **e** V_3,1_^in^ = {x_1_, x_3_, x_6_} and V_3,1_ = {x_8_, x_9_}. The input signal coming from (x_1_, x_3_, x_6_) into V_3,1_ is cyclic with period 12. The SCC V_3,1_ has a unique attractor which is acyclic. **f** *V*_3,2_^in^ = {x_7_} and V_3,2_ = {x_10_, x_11_}. The input signal coming from x_7_ into V_3,2_ is cyclic with period 6. The SCC V_3,2_ has a unique attractor which is acyclic. **g** Table for all the local attractors of subnetworks in the HPFP with the signal [10, 01] in *V*_1,1_. The second column denotes the local attractor [10, 01] of *V*_1,1_ = {*x*_1_, *x*_2_}, where each state in the attractor has its position denoted by the order in the first column. **h** Sequential concatenation of the local attractors in the Table. This yields the unique global attractor of the HPFP, which is cyclic with a period of 12 and has the local attractor 〚10, 01〛 in *V*_1,1_

Let us explain, through an example, how to sequentially concatenate the local attractors of subnetworks in categories of the HPFP in Fig. 3a for the construction of the global attractors of the HPFP. The algorithm for the concatenation is described in detail in Additional file 8. Our explanation is focused on concatenation from the HPFP, so that we do not take into account the external, phenotype, secondary-external and secondary-phenotype nodes, and secondary-phenotype equations. However, when applying our approach to biological networks in the Results section, these were taken into account. The HPFP is given with the fully-simplified update rules in Additional file 7(a). In the following, we describe the sequential concatenation step by step (the details of calculation in each step are given in Additional file 7).

Step 1. Find local attractors in the first category. There exists only one subnetwork *V*_1,1_ = {*x*_1_, *x*_2_} in the first category as it is the unique subnetwork with no input links. Then *V*_1,1_ has the update rules defined by *x*_1_ and *x*_2_ in Additional file 7(a), which yield three local attractors a_〈1〉_ = 〚10, 01〛, a_〈2〉_ = 〚00〛, a_〈3〉_ = 〚11〛 in Additional file 7(b). Here the symbol 〚10, 01〛 denotes a cyclic attractor of length 2 and 〚00〛 a point attractor in Fig. 3b. In the next, we find global attractors containing the cyclic states a_〈1〉_ for *x*_1_ and *x*_2_. Similarly, the process for obtaining global attractors that include a_〈2〉_ and a_〈3〉_ is presented in detail in Additional file 8.

Step 2. Find local attractors in the second category. There exist two subnetworks *V*_2,1_ = {*x*_3_, *x*_4_} and *V*_2,2_ = {*x*_5_, *x*_6_, *x*_7_} in the second category with input signals from the first category.

Step 2-1. Find local attractors of *V*_2,1_. Due to *V*_2,1_^*in*^ = {*x*_1_}, the subnetwork *V*_2,1_ = {*x*_3_, *x*_4_} gets the input signal 〚1, 0〛 generated from the state values of *x*_1_ in the attractor a_〈1〉_. Note that change of the starting value of the input signal while preserving the order (1 and 0 with period 2) cannot affect the local attractors, which is proved in S.Theorem 1 in Additional file 8. Then we have a unique local attractor of *V*_2,1_^*in*^ ∪ *V*_2,1_ = {*x*_1_, *x*_3_, *x*_4_}, which is 〚101, 000, 100, 010〛 in Fig. 3c and Additional file 7(c). Therefore, the local attractor of *V*_2,1_ = {*x*_3_, *x*_4_} becomes 〚01, 00, 00, 10〛 in Fig. 3g.

Step 2-2. Find local attractors of *V*_2,2_. As in Step 2-1, using the update rules for *V*_2,2_ = {*x*_5_, *x*_6_, *x*_7_} with the input signal 〚0, 1〛 generated from *V*_2,2_^*in*^ = {*x*_2_}, we have a unique local attractor of *V*_2,2_^*in*^ ∪ *V*_2,2_, which is 〚0000, 1010, 0101, 1000, 0001, 1001〛 in Fig. 3d and Additional file 7(d). Therefore the local attractor of *V*_2,2_ = {*x*_5_, *x*_6_, *x*_7_} becomes 〚000, 010, 101, 000, 001, 001〛 in Fig. 3g.

Step 3. Find local attractors in the last category. There exist two subnetworks *V*_3,1_ = {*x*_8_, *x*_9_} and *V*_3,2_ = {*x*_10_, *x*_11_} in the last category with input signals from the first and second categories.

Step 3-1. Find local attractors of *V*_3,1_. Using the update rules for *V*_3,1_ = {*x*_8_, *x*_9_} with the input signal 〚100, 001, 100, 010, 100, 000, 100, 011, 100, 000, 100, 010〛 from *V*_3,1_^*in*^ = {*x*_1_, *x*_3_, *x*_6_}, we have a unique attractor of *V*_3,1_^*in*^ ∪ *V*_3,1_, which is cyclic with length 12

$$ \left\llbracket \begin{array}{l}10000,00100,10000,01000,10000,00000,\\ {}10000,01100,10000,00000,10000,01000\end{array}\right\rrbracket $$

in Fig. 3e and Additional file 7(e). Therefore the local attractor of *V*_3,1_ = {*x*_8_, *x*_9_} becomes 〚00〛 in Fig. 3g.

Step 3-2. Local attractors of *V*_3,2_. Using the update rules for *V*_3,2_ = {*x*_10_, *x*_11_} with the input signal 〚0, 0, 1, 0, 1, 1〛 from *V*_3,2_^*in*^ = {*x*_7_}, we have a unique attractor of *V*_3,2_^*in*^ ∪ *V*_3,2_, which is 〚000, 000, 100, 000, 100, 100〛 in Fig. 3f and Additional file 7(f). Therefore the local attractor of *V*_3,2_ = {*x*_10_, *x*_11_} becomes 〚00〛 in Fig. 3g and Additional file 7(f).

Step 4. Construct the table with all the local attractors obtained from a_〈1〉_. We construct the table in Fig. 3g, in which the first column denotes the order of states of each local attractor in the second column to the sixth column. The second column denotes the local attractor 〚10, 01〛 of *V*_1,1_ = {*x*_1_, *x*_2_}. Even though there are no states in the cells of the second column from the order 3, the second column is considered to be filled with states 10 and 01 with a period of 2. For instance, the states in the 3rd and 4th cells are 10 and 01, respectively. Similarly, the third column is filled with states 01, 00, 00 and 10 with a period of 4. Repeating this process completes the table.

Step 5. Construct the global attractor from the table. The concatenation of states in the i-th row of the table becomes the i-th state of the global attractor of the HPFP. Therefore, concatenating states from cells of the table in a row yields the unique global attractor of the HPFP with a period of 12, where the period is the least common multiple of periods of the five local attractors: 2, 4, 6, 1 and 1.

Finally, we obtain the unique global attractor by sequentially concatenating the local attractors that include the local attractor a_〈1〉_ in Fig. 3h and Additional file 7(g), and confirm that the concatenated states become the global attractor by applying the original update rules in Additional file 7(g).

## Results

To demonstrate the effectiveness of our framework in practice, we applied our method to three biological network models for finding attractors responsible for proliferation or apoptosis phenotypes: the first was a Mitogen-activated protein kinase (MAPK) model [6] with 53 nodes and 88 links, the second was a colitis-associated colon cancer (CACC) model [10] with 70 nodes and 152 links and the last was the simplified cancer pathways (SCP) model [9] with 96 nodes and 265 links. In general, Boolean update rules are classified into two types: one is defined with logic functions and the other with threshold functions. The MAPK and CACC networks have update rules that correspond to the first type. The SCP network has update rules corresponding to the second type.

### MAPK network

**Fig. 4:**
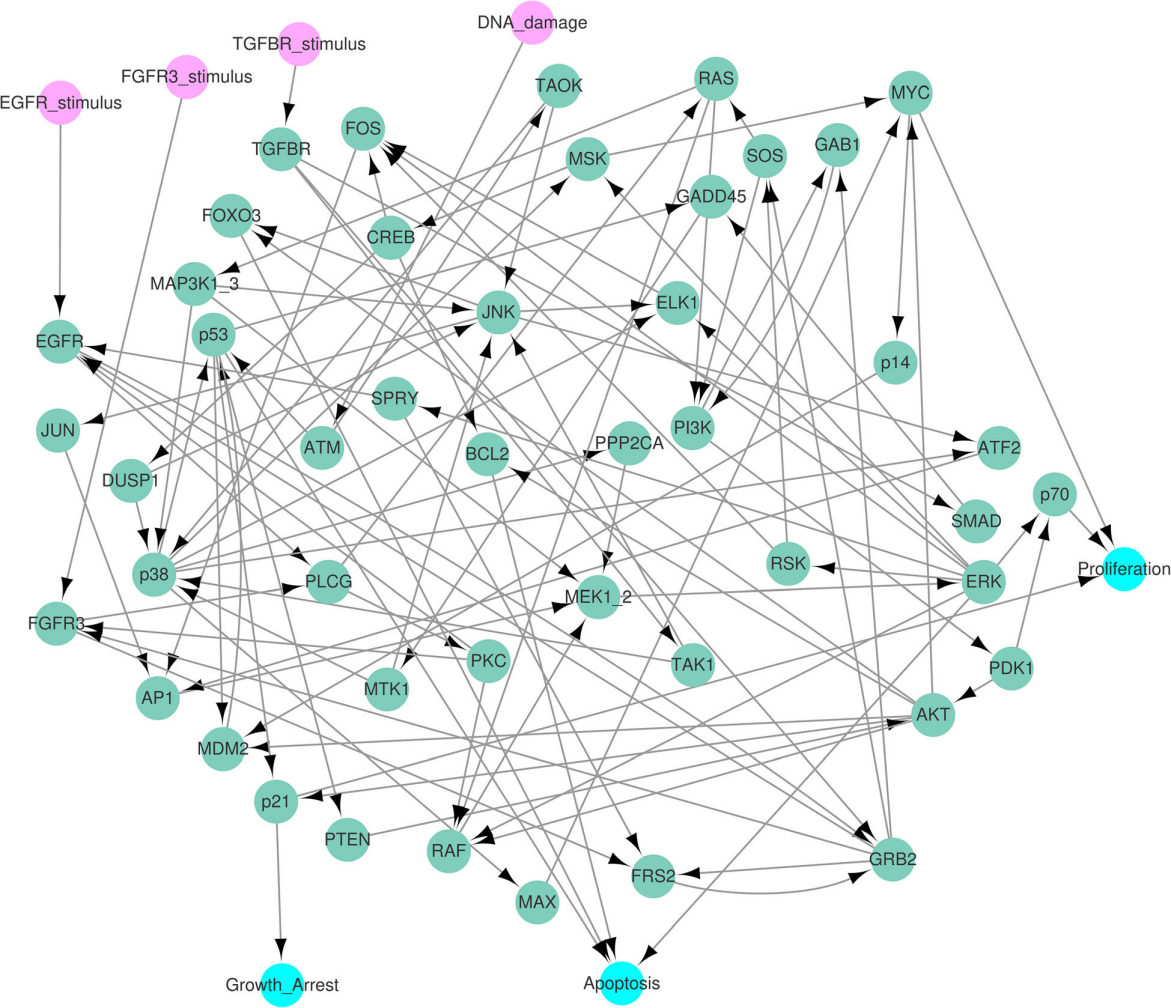
MAPK network. The network denotes a Mitogen-activated protein kinase (MAPK) network with 53 nodes and 88 links in [6]. The *pink* and *blue* nodes denote stimuli and phenotypes, respectively

The MAPK model has four stimuli (DNA damage, TGFBR stimulus, EGFR stimulus and FGFR3 stimulus) and three phenotypes (proliferation, apoptosis and growth arrest) as in Fig. 4. The cascades in the MAPK network are strongly interconnected and the maximum number of nodes of the SCCs in the network is 37(69.8 %) as shown in Additional files 2(a) and (b). Therefore, due to computational complexity, the previous methods [30, 31] based on SCCs is not useful for this MAPK network.

### Proliferation attractors of the MAPK network

**Fig. 5:**
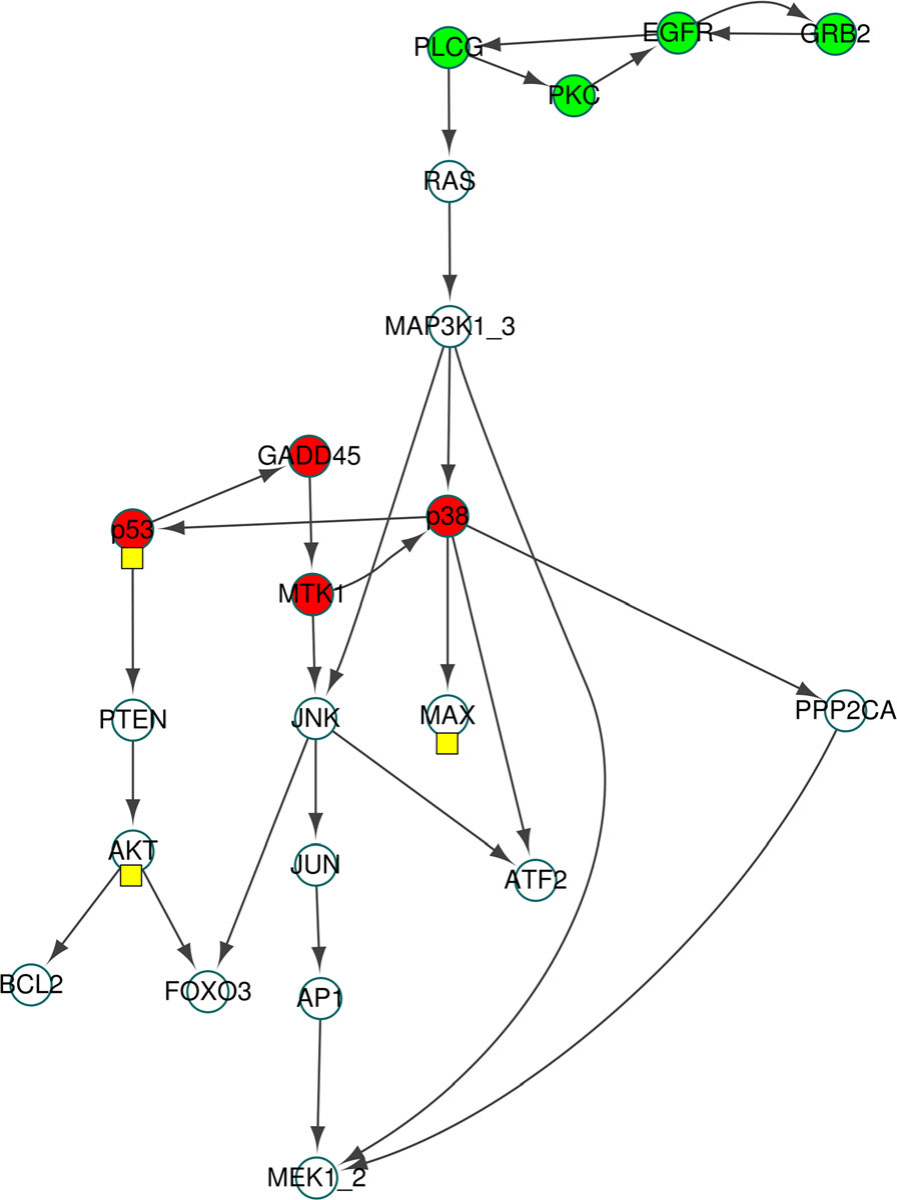
Simplified MAPK network for proliferation attractors. When finding attractors for proliferation in the MAPK model, the original update rules for MAPK network is divided into two parts. The first is fixed state values of ESENs and PSPNs. The second is the simplified update rules for nodes (N) except both ESENs and PSPNs. The network in this figure is the hierarchically partitioned network with the simplified update rules for the nodes N. There exist only two SCCs *V*_1,1_ and *V*_4,1_ that have more than one node. The nodes in *V*_1,1_ and *V*_4,1_ are colored with *green* and *red*, respectively. The *yellow* boxes denote the nodes used in the constraint equations (MAX|AKT = 1, ! AKT & p53 = 0)

In order to find global attractors for proliferation of the MAPK network, we used the same simulation condition r30 in S3 Dataset of [6]: ERK perturbation with setting the values of the four stimuli to zero. Inserting the fixed values (ERK, TGFBR stimulus, EGFR stimulus, FGFR3 stimulus, DNA damage) = (1,0,0,0,0) of the external nodes into the update rules for the MAPK network in Additional file 2(a), we found the external condition: the external, secondary-external nodes (ESENs) and the semi-simplified update rules for nodes except ESENs in Additional file 2(c). Due to the update rule, Proliferation* = p70 & MYC & !p21, inserting the phenotype values (p70, MYC, p21) = (1,1,0) of the phenotype nodes into the semi-simplified update rules, we found the phenotype condition: the phenotype and secondary-phenotype nodes (PSPNs), the fully-simplified update rules for 21 nodes and the two secondary-phenotype equations

$$ \mathrm{MAX}\ \Big|\ \mathrm{A}\mathrm{K}\mathrm{T}=1,\ !\mathrm{A}\mathrm{K}\mathrm{T}\ \&\ \mathrm{p}53=0 $$

in Additional file 2(d). The fully-simplified update rules yield the HPFP for proliferation in Fig. 5 where the HPFP has 8 categories and the SCCs has four nodes at most whereas the SCC in the original network has 37 nodes. The yellow boxes on the three nodes p53, MAX, AKT in Fig. 5 denote the nodes included in the two secondary-phenotype equations, where the three nodes are referred to as “the equation nodes”.

The SCCs with more than one node in the HPFP are *V*_1,1_ = {GRB2, PKC, EGFR, PLCG} and *V*_4,1_ = {p38, p53, GADD45, MTK1}. The fully-simplified update rules for (GRB2, PKC, EGFR, PLCG) yield that *V*_1,1_ has the unique attractor 〚0100, 0000, 0010, 1011, 1101〛 in Additional file 2(e), where the computing time was 0.028871 s by using a PC with 3.6GHz CPU and 32G RAM. The signal coming from {PLCG} in *V*_1,1_ is transmitted to *V*_2,1_ = {RAS} with the formula RAS* = PLCG and the signal from {RAS} becomes the input signal to *V*_3,1_ = {MAP3K1_3} with MAP3K1_3* = RAS. As a result, *V*_3,1_ has a unique attractor 〚1, 1, 0, 0, 0〛. The unique input signal from {MAP3K1_2} to *V*_4,1_ yields a unique attractor of *V*_4,1_, which is the point attractor 〚0000〛 for (p38, p53, GADD45, MTK1) in Additional file 2(f). In this case, the computing time was 0.108352 s. Inserting the state values of the point attractor into the fully-simplified update rules in Additional file 2(d), we have

$$ \mathrm{PTEN}*=\mathrm{p}53=0,\ \mathrm{A}\mathrm{K}\mathrm{T}*=!\mathrm{PTEN}=1,\ \mathrm{MAX}*=\mathrm{p}38=0, $$

and then

$$ \mathrm{MAX}\Big|\mathrm{A}\mathrm{K}\mathrm{T}=1,\ !\mathrm{A}\mathrm{K}\mathrm{T}\&\mathrm{p}53=0. $$

Hence the secondary-phenotype equations were satisfied for the local attractors of the seven subnetworks

*V*_*i*,1_(1 ≤ *i* ≤ 4), *V*_5,1_ = {PTEN}, *V*_5,3_ = {MAX}, *V*_6,1_ = {AKT}.

Note that the remaining eight subnetworks in Fig. 5 do not affect the states of the equation nodes (p53, MAX, AKT). Therefore, we can construct the table in Fig. 3g and found a unique global attractor for proliferation, which is cyclic with a period of 5 in Additional file 2(g). We confirmed that the set of cyclic states becomes a global attractor of the original network by applying the original update rules in Additional file 2(g).

### Apoptosis attractors of the MAPK network

**Fig. 6:**
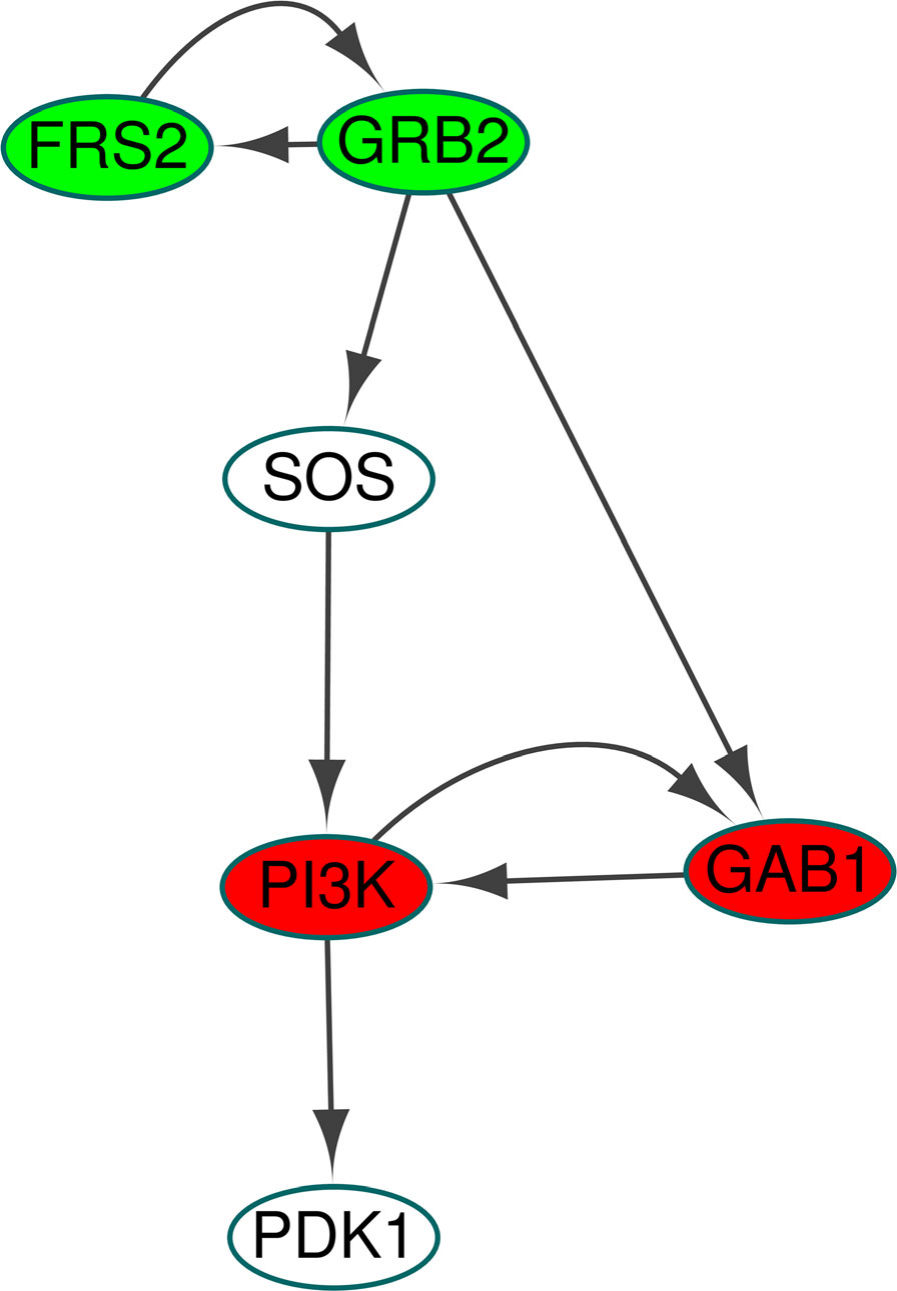
Simplified MAPK network for apoptosis attractors. When finding attractors for apoptosis in the MAPK model, the original update rules for MAPK network is divided into two parts. The first is fixed state values of ESENs and PSPNs. The second is the simplified update rules for nodes (N) except both ESENs and PSPNs. The network in this figure is the hierarchically partitioned network for the nodes N with the simplified update rules. There exist only two SCCs *V*_1,1_ and *V*_3,1_ that have more than one node. The nodes in *V*_1,1_ and *V*_3,1_ are colored with *green* and *red*, respectively. In this case there exist no constraints, so that there are no *yellow* boxes as in Fig. 5

To find global attractors for apoptosis phenotype in the MAPK network, we used the simulation condition r4 in S3 Dataset of [6], which is FGFR3 perturbation with setting the values of the four stimuli to zero. The fully-simplified update rules yield the HPFP for apoptosis in Fig. 6, where the HPFP has 4 categories and each SCC has two nodes at most. We obtained a unique global attractor for apoptosis, which is cyclic with a period of 4 in Additional files 3 and 9.

### CACC network

**Fig. 7:**
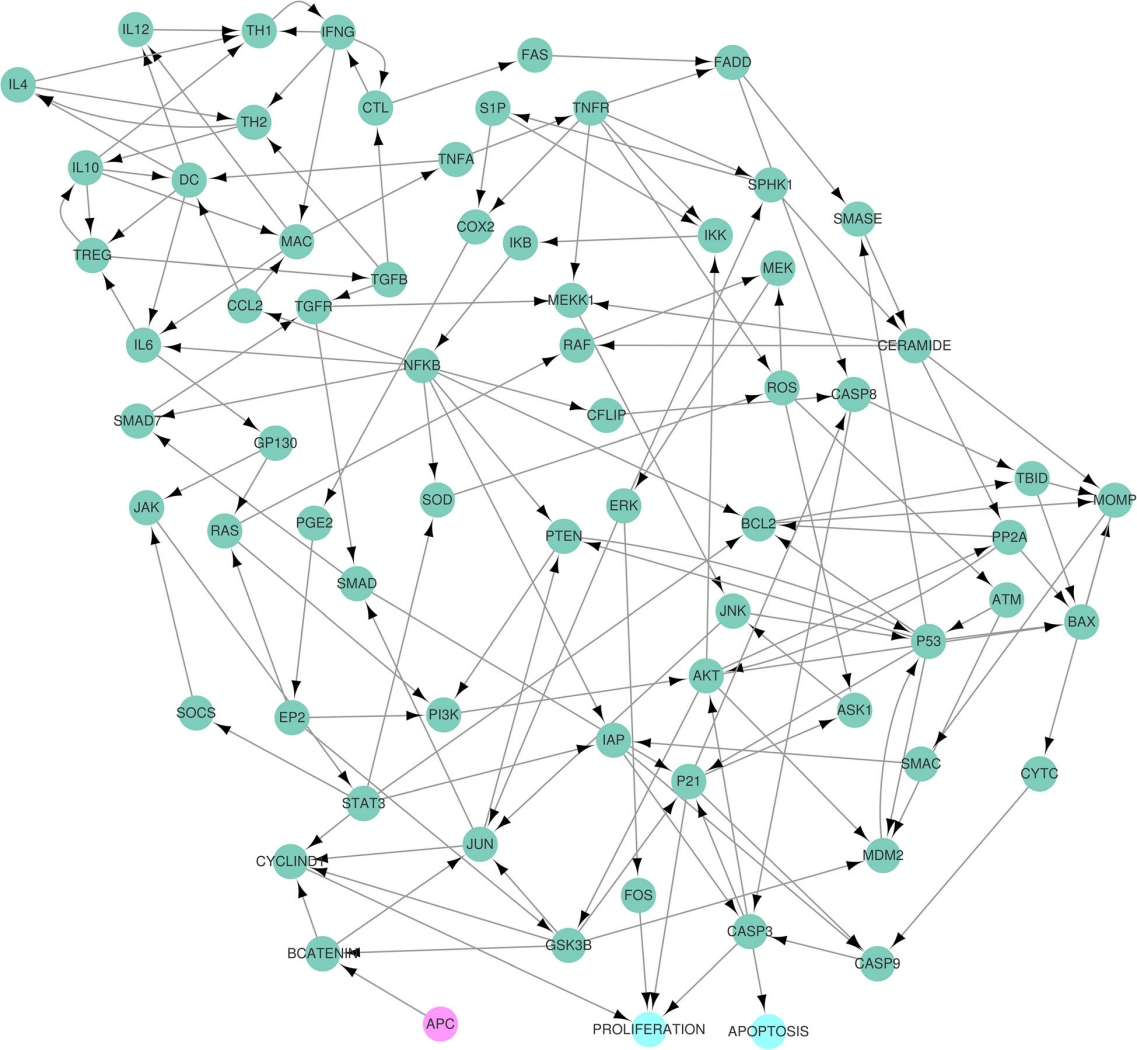
CACC network. The network denotes a colitis-associated colon cancer (CACC) network with 70 nodes and 152 links in [10]. The *pink* circle denotes the input node, the adenomatous polyposis coli (APC) protein that represents premalignant intestinal epithelial cells when consistently activated. The *cyan* nodes denote phenotypes

The CACC model has one input (APC) and two phenotypes (proliferation and apoptosis) as in Fig. 7. The CACC network model is strongly interconnected with the maximum number of nodes of the SCC in the network is 65 (92.9 %) as described in Additional file 4(a) and (b).

### Proliferation attractors of the CACC network

**Fig. 8:**
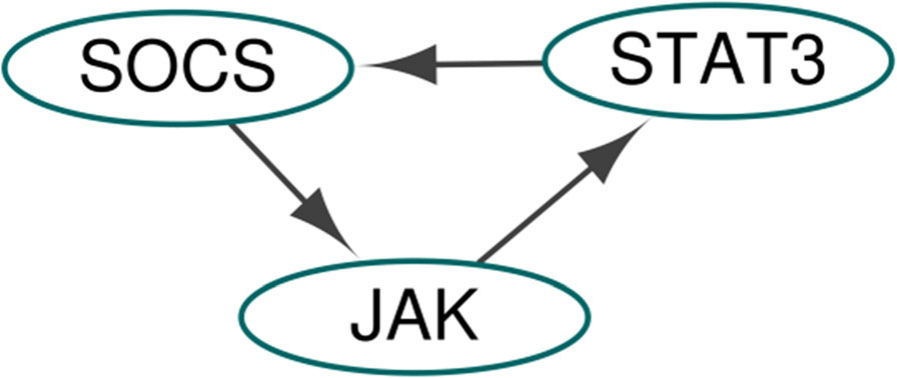
Simplified CACC network for proliferation attractors. The network denotes the simplified CACC network for nodes except ESENs and PSPNs with the simplified update rules when finding attractors for proliferation in the CACC model

Under the strong tumour-promoting microenvironment (fixing DC at ON) for premalignant intestinal epithelial cells (fixing APC at ON) as in [10], we applied the proposed method to find global attractors for proliferation in the CACC network with the update rules in Additional file 4(a). Inserting the external values (DC, APC) = (1,1) of the external nodes into the CACC update rules in Additional file 4(a), we found the external condition: the external and secondary-external nodes (ESENs) and the semi-simplified update rules for nodes except ESENs in Additional file 4(c). Due to Proliferation* = (FOS & CYCLIND1) & !(P21 | CASP3) with the secondary-external value FOS = 1, inserting the values (CYCLIND1, P21, CASP3) = (1,0,0) into the semi-simplified update rules, we found the phenotype condition: the phenotype, secondary-phenotype nodes (PSPNs), the fully-simplified update rules for three nodes (SOCS, STAT3, JAK) and no secondary-phenotype equation in Additional file 4(d). The fully-simplified update rules yield the HPFP for proliferation in Fig. 8 and the HPFP has one category with one SCC *V*_1,1_, which has three nodes. The fully-simplified update rules for the three nodes (SOCS, STAT3, JAK) yield that *V*_1,1_ has two attractors

**Fig. 9:**
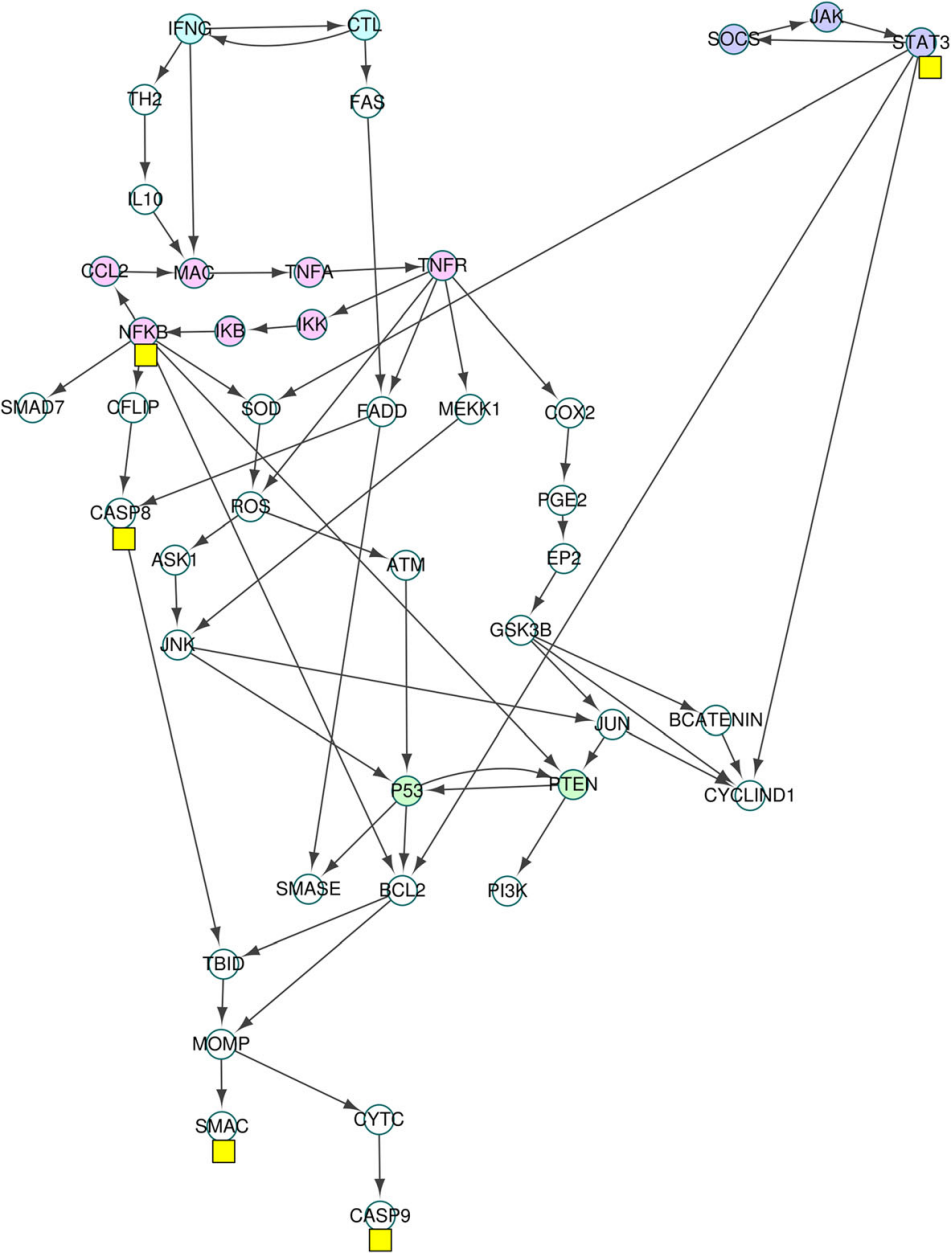
Simplified CACC network for apoptosis attractors. When finding attractors for apoptosis in the CACC model, the network denotes the hierarchically partitioned CACC network for nodes except ESENs and PSPNs with the simplified update rules. There exist four SCCs with more than one node, which are represented with different colors:*V*_1,1_ = {IFNG, CTL}, *V*_1,2_ = {SOCS, JAK, STAT3}, *V*_4,1_ = {CCL2, MAC, TNFA, TNFR, IKK, IKB, NFKB}and *V*_10,1_ = {P53, PTEN}. The yellow boxes denote the nodes used in the constraint equations ((NFKB|STAT3)&(~ SMAC) = 0, CASP8|CASP9 = 1)

$$  010,\ 101,\  111,\ 110,\ 100,\ 000,\ 001,\ 011 $$

in Additional file 4(e). Therefore we found two global attractors for proliferation in Additional file 4(f) and (g), which are confirmed by applying the CACC update rules in Additional file 4(f) and (g).

### Apoptosis attractors of the CACC network

Under the same condition for proliferation attractors of the CACC network above, the fully-simplified update rules yield the HPFP for apoptosis in Fig. 9. We applied our method to the CACC network and found that there exists no global attractor for apoptosis in the CACC network in Additional files 5 and 10. Since the simulation condition (DC, APC) = (1,1) denotes the strong tumour-promoting microenvironment (fixing DC at ON) for premalignant intestinal epithelial cells (fixing APC at ON), the non-existence of global attractors for apoptosis can be expected.

### SCP network

**Fig. 10:**
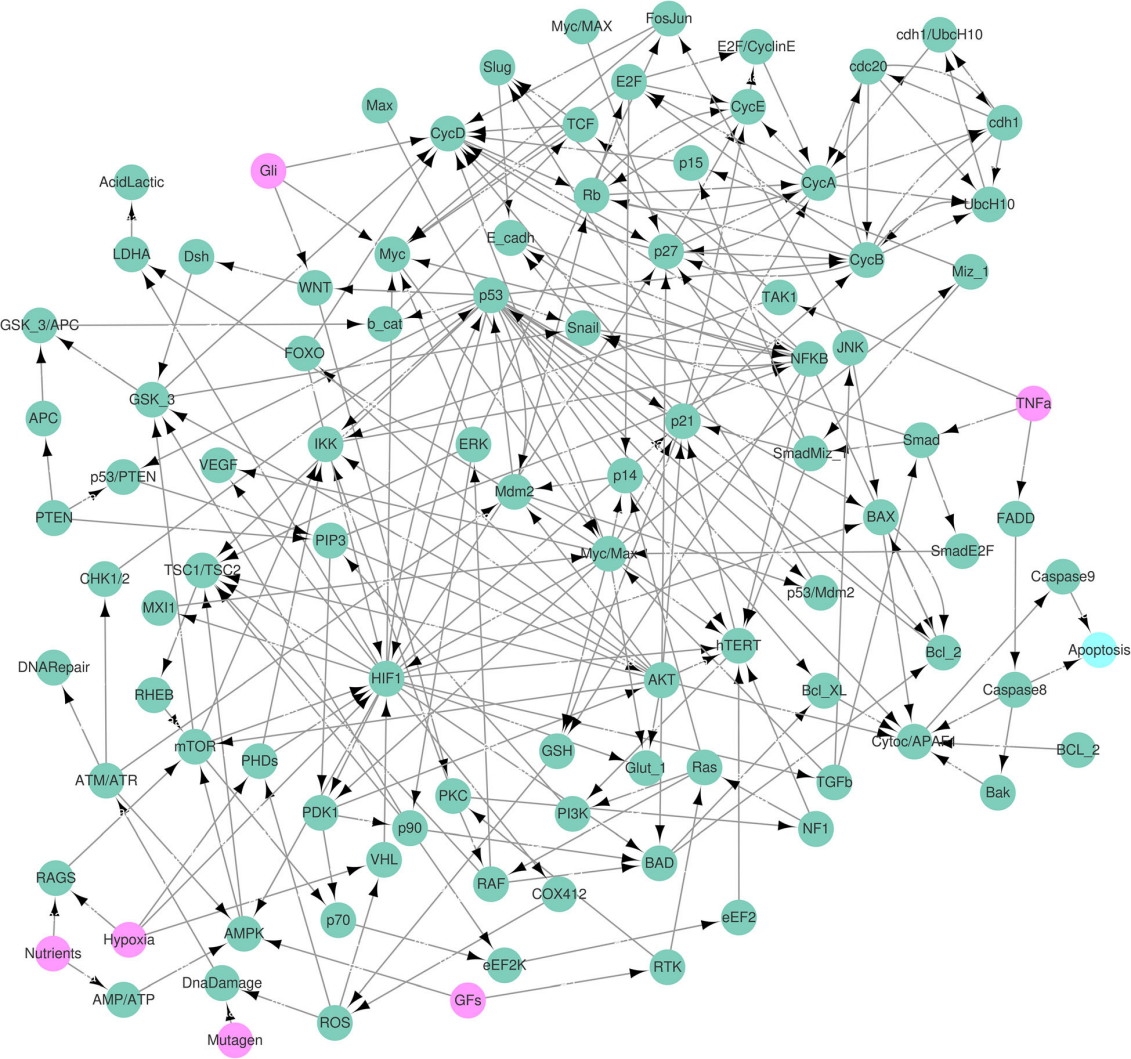
SCP network. The network denotes a simplified cancer pathways (SCP) network with 96 nodes and 265 links in [9]. The *pink* and *cyan* nodes denote input and apoptosis phenotype, respectively

The simplified cancer pathway model has six inputs (Mutagen, GFs, Nutrients, TNfa, Hypoxia and Gli) and one phenotype (apoptosis) as shown in Fig. 10. The cascades in the original network are strongly interconnected with the maximum number of nodes of the SCC is 68 (70.8 %) as described in Additional file 6(a) and (b). The state vector (Mutagen, GFs, Nutrients, TNfa, Hypoxia) = (0,0,1,0,1) describes the normoxic microenvironment with the plenty of nutrients and growth factors [9].

We considered the apoptosis phenotype under the same microenvironment in [9]. Inserting the external values (Mutagen, GFs, Nutrients, TNfa, Hypoxia, Gli) = (0,0,1,0,1,0) into the SCP update rules in Additional file 6(a), we found the external condition: the external, secondary-external nodes (ESENs) and the semi-simplified update rules for nodes except ESENs in Additional file 6(c). The secondary-external value Caspase8 = 0 yields

$$ \mathrm{Apoptosis}*=\mathrm{s}\mathrm{g}\mathrm{n}\left[\mathrm{Caspase}8+\mathrm{Caspase}9\right] = \mathrm{s}\mathrm{g}\mathrm{n}\left[\mathrm{Caspase}9\right]. $$

Note that Apoptosis =1 if and only if Caspase9 = 1, which is also equivalent to (Caspase9, Cytoc/APAF1) = (1,1) and the constraint inequality 0 < -AKT + p53-BCL_2-Bcl_XL since Caspase9* = Cytoc/APAF1 and Cytoc/APAF1* = sgn(-AKT + p53-BCL_2-Bcl_XL). As a result of the external condition, the set of ESENs satisfies the constraint inequality and becomes the set of all nodes in the SCP network in Additional file 6(c). Then there are no PSPNs, no secondary-phenotype inequalities and no fully-simplified update rules.

Therefore the vector of the fixed values of the 96 nodes (ESENs) is the unique attractor for apoptosis under the microenvironment. We confirmed that the concatenated state vector becomes the global attractor by applying the SCP update rules to Additional file 6(e).

### Resolving the two problems: large size and strong interconnection

We summarized in Table 1 the aforementioned results obtained for the MAPK, CACC and SCP networks. The three biological networks have a large number of nodes that are strongly interconnected. The first two networks have nodes for representing proliferation and apoptosis phenotypes, but the last has a node for only apoptosis.

**Table 1:** The symbol #A denotes the number of A. And the symbol Max #Nodes in SCCs denotes the maximum of the numbers of nodes in strongly connected components

	Original network		HPFP for nodes except ESENs and PSPNs				
	#Nodes	Max #Nodes in SCCs	Phenotype	#External and phenotype nodes	#Categories	#SCCs	Max #Nodes in SCCs
MAPK	53	37 (69.8 %)	Proliferation	8	8	15	4
			Apoptosis	9	4	4	2
CACC	70	65 (92.9 %)	Proliferation	6	1	1	3
			Apoptosis	3	15	33	7
SCP	97	68 (70.8 %)	Apoptosis	8	0	0	0

The column with the title #External and phenotype nodes in Table 1 shows that the number of nodes for a given external environment and a phenotype of interest are independent of the size and degree of interconnection. We found that the number of SCCs in the HPFP is approximately proportional to the number of categories as the number of categories gets increased. Even if the original networks are strongly interconnected and the number of categories and that of SCCs in the HPFP are high, the number of nodes in SCCs in the HPFP is small enough such that full search of the state space can be performed to find local attractors and therefore we can find global attractors for the phenotype in the original networks by concatenating the local attractors.

In particular, in the case of the SCP network, the set of ESENs becomes the set of all the 97 nodes in the SCP network with no secondary-phenotype inequalities, which explains why the number of categories, that of SCCs and the maximum are all zero in Table 1. The SCP network does not include a node for proliferation, thus we could not find attractors for proliferation phenotype.

### Comparison of our method with the reduction and random sampling methods

Previous methods based on SCCs for finding all global attractors cannot be applied to the three networks in Table 1 due to the large size of networks. To deal with such a problem, two methods are usually used: reduction method and random sampling method, which were compared with our method for the MAPK, CACC and SCP networks.

Under the condition r30 in S3 Dataset of [6] used in the Proliferation attractors of the MAPK network section, the reduction method yields two attractors for proliferation phenotype, which are cyclic with a period of 6 in S3 Dataset of [6]. However, we found that the MAPK update rules under the same condition result in the unique attractor for proliferation, which is cyclic with a period of 5 in Additional file 2(g). Such different results show that the reduced network obtained by the reduction method does not preserve the attractors of the original network, even though the periodic property of attractors is preserved. In addition, under the condition r4 in S3 Dataset of [6], such difference was also found between S3 Dataset of [6] and Additional file 3(f).

Reduced update rules are given for a reduced CACC network in [10] and we used the update rules to find attractors for proliferation of the reduced CACC network in Additional file 11. We compared the proliferation attractors obtained by the reduced update rules with the proliferation attractors obtained from the original CACC network by using the proposed method. As a result, we did not find such a difference between S3 Dataset of [6] and Additional file 3(f). However, the reduced method has a disadvantage that the attractors obtained by reduced update rules for reduced networks do not have the information on states of all nodes in the original networks.

Under the condition (Mutagen, GFs, Nutrients, TNfa, Hypoxia) = (0,0,1,0,1) in S1 Dataset of [9], the random sampling method and our method yield a unique attractor for apoptosis phenotype, which is a fixed point attractor in S1 Dataset of [9] and Additional file 6(e). Even though a random sampling method (i.e. randomly sampling the initial states and tracing the converging state trajectories) can provide an estimate of global attractors of large-size networks while compromising the computational complexity, such an approximation cannot guarantee to find all global attractors for a phenotype of interest. However, our method can always guarantee the full search result even for a large network.

### Validation of the proposed algorithm

To show that all attractors in a given network can be found by applying our method, we applied our method to two Boolean networks with known attractors. The first network is shown in Fig. 3a and it has 11 nodes with the update rules in Additional file 7(a), where the network has neither input nor output nodes. This network is suitable for the validation of concatenating local attractors obtained from HPFP without considering ESENs and PSPNs. By applying a full search algorithm to this network, we could find all attractors and, as a result, we confirmed that these attractors are exactly the same as we found by applying our method (see Additional file 12 for details).

For the validation of PSPNs as well as the concatenation, the second network is adopted from [10] and it has 21 nodes. This network has two cyclic attractors of length 2 and 6, both representing cell proliferation, as shown in Fig. 3c and Supplementary Table S6 in [10]. To find out all attractors representing cell proliferation in this network model, we have applied our method to this network and could obtain the same attractors (see Additional file 12 for details)**,** as shown in Fig. 3c and Supplementary Table S6 in [10].

## Discussion

Previous approaches of partitioning a large Boolean network model to resolve the computational complexity issue preserve the regulatory links of the original network to identify all the attractor states from the partitioned network. The primary point we noticed is that we have to simplify the state update rules and reduce the regulatory links to obtain small subnetworks of practically computable size. For this purpose, we focused only on a set of attractors for a particular phenotype of interest and developed a novel algorithm that can efficiently find out the attractor states from the hierarchically partitioned subnetworks obtained by simplifying the state update rules and replacing some regulatory links with constraint equations while preserving the particular attractor states. In contrast with the previous approaches, the proposed approach can result in small and simplified subnetworks of computable size. An important point is that we can always find out all the attractors corresponding to the phenotype of interest in the original large network by sequentially concatenating the local attractors that are obtained from the hierarchically partitioned subnetworks.

The limitation of our approach is that we cannot find out all attractors at the same time using this approach. However, in many practical case studies, only a few particular phenotypes are of interest and therefore, by applying the proposed approach to each particular phenotype, we can find out all attractor states of interest. Another limitation is that our approach is based on synchronous update rules, so it is currently not applicable to the Boolean network models based on asynchronous update rules. This remains as a future study.

From the case studies where we applied our approach to the three large biological network examples as well as small and medium size networks (Additional files 12 and 13), we found that the resulting subnetworks (i.e. SCCs) are composed of seven nodes at most. Of course, we cannot guarantee such a small size subnetwork in all cases, but we can always obtain much smaller subnetworks compared to previous approaches since our approach simplifies the state update rules in a practical way. Moreover, we can further reduce the subnetwork size if any other biological information on the molecular state of a node in the converging phenotypic feature is available. As there are many other biological networks that are different from the networks employed in this study, it remains as a future study to further investigate the power of our method by using extensive simulation-based analysis of synthetic networks with respect to various topological properties such as different size, level of interconnections, etc.

When we hierarchically partition a network, we simplify the state update rules by considering the fixed state values of marker nodes in the attractor states. As a result, the state space after the hierarchical partitioning becomes a subset of the state space of the original network. So, we cannot measure the basin of attraction to the particular phenotype of the original network in our framework. However, some modification of our framework might be able to resolve the problem. This also remains as a future study.

## Conclusions

Although Boolean network modeling is becoming popular in modeling large-scale biological regulatory networks, looking for attractors for converging state analysis is still challenging for large networks due to computational complexity. There have been some attempts to resolve this problem by partitioning the large network into smaller subnetworks and reconstructing the global attractors by concatenating the local attractors obtained from each subnetwork, but the resultant subnetworks were still too large in most cases and therefore not much useful in practice. So, in this study, we have developed a novel approach of identifying a set of global attractors for a particular phenotype of interest by hierarchically partitioning the original large network such that the resulting subnetworks are small enough to guarantee that the full search of the local attractors of them is possible. We have applied the proposed method to several biological networks and confirmed its usefulness.

Throughout the hierarchical partitioning, we can obtain the hierarchical partitioned structure of the original network with the fixed state values of some nodes. Such structural information on the network might be also useful in identifying certain target nodes to control the phenotypic behavior of a biological network. For instance, we can use this information to find out control target nodes, the perturbation of which results in preventing the convergence of the dynamical network state to the particular attractor state of interest. This is an important subject for a future study.

## Additional files

Additional file 1: Definition of external and phenotype nodes. (PDF 211 kb)
Additional file 2: Attractors for proliferation in the Mitogen-activated protein kinase (MAPK) model. (XLSX 60 kb)
Additional file 3: Attractors for apoptosis in the Mitogen-activated protein kinase (MAPK) model. (XLSX 47 kb)
Additional file 4: Attractors for proliferation in the colitis-associated colon cancer (CACC) model. (XLSX 61 kb)
Additional file 5: Attractors for apoptosis in the colitis-associated colon cancer (CACC) model. (XLSX 107 kb)
Additional file 6: Attractors for apoptosis in the simplified cancer pathways (SCP) model. (XLSX 53 kb)
Additional file 7: Example of how to find the phenotype attractors in HPFP. (XLSX 73 kb)
Additional file 8: Explanation of how to concatenate the local attractors of subnetworks in the HPFP. (PDF 1254 kb)
Additional file 9: Attractors for apoptosis in the MAPK network. (PDF 189 kb)
Additional file 10: Attractors for apoptosis in the CACC network. (PDF 193 kb)
Additional file 11: Attractors for proliferation in the reduced CACC model. (XLSX 38 kb)
Additional file 12: Validation of the proposed algorithm for two small and medium networks. (PDF 158 kb)
Additional file 13: Attractor in the logical T-cell signaling model. (XLSX 19 kb)

## Abbreviations

CACC: Colitis-associated colon cancer
ESENS: External and secondary-external nodes
HPFP: The hierarchical partition for the phenotype
MAPK: Mitogen-activated protein kinase
PSPNs: Phenotype and secondary-phenotype nodes
SCC: Strongly connected component
SCP: Simplified cancer pathways
