# Supplementary material for: An efficient algorithm for identifying primary phenotype attractors of a large-scale Boolean network
Source: BMC Syst Biol. 2016 Oct 7;10:95. doi: 10.1186/s12918-016-0338-4 (PMC5055661; doi:10.1186/s12918-016-0338-4)
Supplement: Additional file 1: — Definition of external and phenotype nodes. (PDF 211 kb) [file 12918_2016_338_MOESM1_ESM.pdf]

# An Efficient Algorithm for Identifying Primary Phenotype Attractors of a Large-Scale Boolean Network

**Sang-Mok Choo[1] and Kwang-Hyun Cho[2,*]**

[1]Department of Mathematics, University of Ulsan, Ulsan 44610, Republic of Korea

[2]Department of Bio and Brain Engineering, Korea Advanced Institute of Science and Technology (KAIST), Daejeon 34141, Republic of Korea

# Supporting information

## Definition of terminology

[*] Corresponding author, E-mail: ckh@kaist.ac.kr, Phone: +82-42-350-4325, Fax: +82-42-350-4310, Web: http://sbie.kaist.ac.kr

Assume that an external environment like external stimulus and node perturbation is given to a synchronous Boolean network. In addition, it is assumed that the Boolean network has a node for representing one phenotype of interest.

The state value of every perturbed node is fixed, which means that the state value is not updated by the Boolean update rules. Then the update equation for the perturbed node is removed from the Boolean update rules, so that the links connected to the perturbed node are removed from the network. Nodes and values for representing perturbation and stimuli are referred to as "external nodes and values", respectively.

Putting the external values into the system of update equations, we can obtain fixed values of some other nodes, which are referred to as "secondary-external nodes and values". As a result, the update rules are divided into two parts. The first is the set of external and secondary-external nodes (ESENs) with their values and the other is the update rules for all nodes except ESENs, which are referred to as "the semi-simplified update rules".

If a node PT for representing a phenotype (for example, PT=Proliferation) in the network is updated by the Boolean update rule $\mathrm{PT}^* = f\left(x_1, \cdots, x_k\right)$, then the independent variables $x_i\left(1 \le i \le k\right)$ are referred to as the "phenotype nodes". Here the symbol * means the next time step. The fixed state values $x_i = x_i^{fixed}$ are also referred to as the "phenotype values" if the equation $1 = f\left(x_1, \cdots, x_k\right)$ has the unique solution $x_i = x_i^{fixed}$. Therefore the "phenotype attractors" can be defined as the attractors in which the phenotype nodes have the phenotype values.

Putting the phenotype values into the semi-simplified update rules, we can obtain both fixed values of some other nodes and a system of equations, which are equivalent to the condition $1 = f\left(x_1, \cdots, x_k\right)$ and referred to as "secondary-phenotype nodes, values and equations". Then the secondary-phenotype equations must be satisfied when finding the phenotype attractors and the links connected to phenotype and secondary-phenotype nodes can be removed in the network due to the secondary-phenotype equations.

Therefore the original update rules are divided into three parts. The first part is the nodes (ESENs and PSPNs) with fixed state values and the second is the constraint equations (the secondary-phenotype equations). The third is a new update rules for all nodes except ESENs and PSPNs. The new update rules are referred to as "the fully-simplified update rules".

**S.Example 1**. The MAPK model [6] in the main text has four stimuli (DNA damage, TGFBR stimulus, EGFR stimulus, FGFR3 stimulus) and three phenotypes (proliferation, apoptosis, growth arrest). Assume that the MAPK model is considered under ERK perturbation with setting values of the four stimuli to zero, which is the simulation condition r30 in S3 Dataset of [6]. In this case, we refer to the five nodes (DNA damage, TGFBR stimulus, EGFR stimulus, FGFR3 stimulus, ERK) as the external nodes.

Putting the external values (TGFBR stimulus, EGFR stimulus, FGFR3 stimulus, DNA damage, ERK)=(0,0,0,0,1) into

the system of update equations, we can obtain fixed values of the nodes (Apoptosis,ATM,CREB,DUSP1,ELK1,FGFR3,FOS,FRS2,MSK,RAF,RSK,SMAD,SOS,SPRY,TAK1,TAOK,TGFBR),which become the secondary-external nodes. Here the fixed values of ESENs and the semi-simplified update rules are in Additional file 2(c).

**S.Example 2**. The state of proliferation in the MAPK model is updated by the logical function Proliferation*=p70&MYC&!p21 in Additional file 2(a) and then the phenotype nodes are p70, MYC and p21 with the phenotype values (p70, MYC, p21)=(1,1,0).

**S.Example 3**. Applying the phenotype values in S.Example 2 to the update rules in the MAPK model, we have the system of equations

1=p70*=PDK1 & ERK,

1=MYC*=(MSK&MAX)|(MSK&AKT),

0=p21*=!AKT&p53.

Due to the secondary-external values, the equation 1=p70*=PDK1 & ERK is equivalent to

1=p70*=PDK1 & ERK=PDK1&1=PDK1

or equivalent to

(p70, PDK1)=(1,1) and PDK1*=PI3K

or equivalent to

(p70, PDK1, PI3K)=(1,1,1) and PI3K*=GAB1|(RAS&SOS)*=GAB1|(RAS&0)=GAB1

or equivalent to

(p70, PDK1, PI3K, GAB1)=(1,1,1,1) and GAB1*= GRB2 | PI3K= GRB2 | 1=1.

Then using the secondary-external values (ERK, SOS)=(1,0), we have

1=p70*=PDK1 & ERK if and only if (p70, PDK1, PI3K, GAB1)=(1,1,1,1).

Here the nodes (PDK1, PI3K, GAB1) become secondary-phenotype nodes with values (1,1,1).

Similarly, using the secondary-external value MSK=1, we have

1=MYC*=(MSK&MAX)|(MSK&AKT)= (1&MAX)|(1&AKT)= MAX|AKT,

0=p21*=!AKT&p53.

The other secondary-external and phenotype values are listed in Additional files 2(c) and 2(d).

Therefore the phenotype attractors obtained from the fully-simplified update rules in Additional file 2(d) must satisfy the two secondary-phenotype equations

MAX|AKT=1, !AKT&p53=0.

**S.Remark 1**. Assume that a node for representing the phenotype in a Boolean network is updated by a threshold function like

$$Apoptosis^* = sgn[Caspase8 + Caspase9]$$

where the symbol sgn denotes the sign function with value 0 for Caspase8+Caspase9 $\leq 0$ and value 1 for Caspase8+Caspase9$>0$. Then constraint equations are replaced by constraint inequalities. For example, using the secondary-external value Caspase8=0, we have Apoptosis=1 if and only if Caspase9=1, which is also equivalent to (Caspase9, Cytoc/APAF1)=(1,1) and the constraint inequality 0<-AKT+p53-BCL_2-Bcl_XL.

**S.Remark 2**. The links to the external, secondary-external, phenotype and secondary-phenotype nodes are removed from the network. However there is a difference between the external and phenotype nodes. The values of the phenotype nodes are fixed by using the system of update equations and then produce some secondary-phenotype equations. However, the values of the external nodes are fixed regardless of the update rules and then do not produce secondary-phenotype equations.
