# Supplementary material for: An efficient algorithm for identifying primary phenotype attractors of a large-scale Boolean network
Source: BMC Syst Biol. 2016 Oct 7;10:95. doi: 10.1186/s12918-016-0338-4 (PMC5055661; doi:10.1186/s12918-016-0338-4)
Supplement: Additional file 8: — Explanation of how to concatenate the local attractors of subnetworks in the HPFP. (PDF 1254 kb) [file 12918_2016_338_MOESM8_ESM.pdf]

An Efficient Algorithm for Identifying Primary Phenotype Attractors of a Large-Scale Boolean Network

Sang-Mok Choo[1] and Kwang-Hyun Cho[2,*]

[1]Department of Mathematics, University of Ulsan, Ulsan 44610, Republic of Korea

[2]Department of Bio and Brain Engineering, Korea Advanced Institute of Science and Technology (KAIST), Daejeon 34141, Republic of Korea

Supporting information

How to concatenate the local attractors of subnetworks in the HPFP

[*] Corresponding author, E-mail: ckh@kaist.ac.kr, Phone: +82-42-350-4325, Fax: +82-42-350-4310, Web: http://sbie.kaist.ac.kr

In the Method section, we used a simple example for how to sequentially concatenate local attractors. In this supplementary, we explain the concatenation in general.

Let $V=\{x_1,\cdots,x_n\}$ denote the set of nodes for some positive integer n and x_i^t represent the state of the node x_i at time step t for each positive integer t, where the state takes the value of either 0(inactive) or 1(active). The states of all nodes are updated at the same time according to the last states of the Boolean network. The updating rules are

$$x_i^{t+1}=f_i\left(x_1^t,\cdots,x_n^t\right),\ i=1,2,\cdots n,$$

which are written in the vector form as

$$\mathbb{X}^{t+1}=f\left(\mathbb{X}^t\right).$$

We define a cycle of length $p\geq 1$ as the sequence $[\![a^0,\cdots,a^{p-1}]\!]$ such that

$a^t\left(0\leq t\leq p-1\right)$ are pairwise distinct

and

$$\left(f\left(a^0\right),\cdots,f\left(a^{p-2}\right),f\left(a^{p-1}\right)\right)=\left(a^1,\cdots,a^{p-1},a^0\right).$$

For convenience we will use the symbol $\left(a^0,\cdots,a^{p-1}\right)$ instead of the attractor $[\![a^0,\cdots,a^{p-1}]\!]$ in some situation. Fixed points $\left(p=1\right)$ and cycles $\left(p>1\right)$ are called attractors of the Boolean network. For simplicity we call the attractors "attractors of V".

Consider the subsets $V_1,\cdots,V_\rho$ of $V=\{x_1,\cdots,x_n\}$ that satisfy

$$V=V_1\cup\cdots\cup V_\rho,\ V_i\cap V_j=\varnothing\left(1\leq i,j\leq\rho,\ i\neq j\right).$$

Then the set $\{V_1,\cdots,V_\rho\}$ is a partition of V where V_i is denoted by

$$V_i=\left\{x_{i,1},\cdots,x_{i,\theta_i}\right\}.$$

Denote the set of every node in $V-V_i$ that has at least an outgoing edge to some of nodes in V_i by

$$V_i^{in}=\left\{x_{i,1}^{in},\cdots,x_{i,\rho_i}^{in}\right\}.$$

Similarly, denote the set of every node in V_i that has at least an outgoing edge to some of nodes in $V-V_i$ by

$$V_i^{out}=\left\{x_{i,1}^{out},\cdots,x_{i,\xi_i}^{out}\right\}.$$

The update rules for $\left(V_i^{in},V_i\right)$ become

$$x_{i,j}^{t+1}=f_{i,j}\left(x_1^t,\cdots,x_n^t\right)$$

where $1\leq j\leq\theta_i$ and $f_{i,j}\in\{f_1,\cdots,f_n\}$. Since all nodes in $V-\left(V_i^{in}\cup V_i\right)$ do not appear in the expression defining the functions $f_{i,j}$, we can equivalently write $x_{i,j}^{t+1}=f_{i,j}\left(x_1^t,\cdots,x_n^t\right)$ as

$$x_{i,j}^{t+1} = \tilde{f}_{i,j}\left(x_{i,1}^{in,t},\cdots,x_{i,\rho_i}^{in,t},x_{i,1}^{t},\cdots,x_{i,\theta_i}^{t}\right) \text{ or } \mathbb{X}_i^{t+1} = \tilde{\mathrm{f}}_i\left(\mathbb{X}_i^{in,t},\mathbb{X}_i^{t}\right).$$

Hence the nodes $x_{i,j}^{in,t}$ in V_i^{in} can be considered to generate input signal into $\mathbb{X}_i^{t+1} = \tilde{\mathrm{f}}_i\left(\mathbb{X}_i^{in,t},\mathbb{X}_i^{t}\right)$ and so we call each node in V_i^{in} "an input node to V_i" or "an input signal into V_i". Similarly, we call each node in V_i^{out} "an output node from V_i". The pair $\left(V_i^{in},V_i\right)$ becomes a subnetwork with the update rules $\mathbb{X}_i^{t+1} = \tilde{\mathrm{f}}_i\left(\mathbb{X}_i^{in,t},\mathbb{X}_i^{t}\right)$.

Now we are ready to define the hierarchical partition.

S.Definition 1. We call a partition $\bigcup_{i=1}^{m}\left\{V_{i,1},\cdots,V_{i,\tau_i}\right\}$ of V "a hierarchical partition of V" for some positive integer m if the partition satisfies the three conditions.

(C1) there are no input nodes to $V_{1,k}$ and no interaction between $V_{1,k}$ and V_{1,k_1} $\left(1 \le k,k_1 \le \tau_1, k \ne k_1\right)$.

(C2) the set of all input nodes to $\bigcup_{k=1}^{\tau_i} V_{i,k}\left(2 \le i \le m\right)$ is a nonempty subset of the set of all output nodes from $\bigcup_{\ell=1}^{i-1}\left(\bigcup_{k=1}^{\tau_\ell} V_{\ell,k}\right)$. There is also no interaction between $V_{i,k}$ and V_{i,k_i} $\left(i \le k,k_i \le \tau_i, k \ne k_i\right)$.

(C3) there are no output nodes from $V_{m,k}\left(1 \le k \le \tau_m\right)$

The set $V_{1.k}$ is called "the $(1,k)$ subnetwork with the update rules $\mathbb{X}_{1,k}^{t+1} = \tilde{\mathrm{f}}_{1,k}\left(\mathbb{X}_{1,k}^{t}\right)$ and $\left\{V_{i,1},\cdots,V_{i,\tau_i}\right\}$ "the i-th category". The pair $\left(V_{i,k}^{in},V_{i,k}\right)$ becomes "the (i,k) subnetwork" with the update rules $\mathbb{X}_{i,k}^{t+1} = \tilde{\mathrm{f}}_{i,k}\left(\mathbb{X}_{i,k}^{in,t},\mathbb{X}_{i,k}^{t}\right)$ for $i \ge 2$. Note that all $V_{i,k}$ are strongly connected components and there exists a unique hierarchical partition of a given network.

S.Example 1. In the case of S.Fig. 1. below, there exists a subnetwork $V_{1,1}$ which has the empty set $V_{1,1}^{in}$.

The subnetworks and update rules in the first, second, third and last categories are denoted by

$$V_{1,1} = \left\{x_6,x_7,x_8,x_9\right\}, \qquad \mathbb{X}_{1,1}^{t+1} = \tilde{\mathrm{f}}_{1,1}\left(\mathbb{X}_{1,1}^{t}\right) = \tilde{\mathrm{f}}_{1,1}\left(x_6^t,x_7^t,x_8^t,x_9^t\right)$$

$$\left(V_{2,1}^{in},V_{2,1}\right) = \left(\left\{x_7\right\},\left\{x_3,x_4,x_5\right\}\right), \qquad \mathbb{X}_{2,1}^{t+1} = \tilde{\mathrm{f}}_{2,1}\left(\mathbb{X}_{2,1}^{in,t},\mathbb{X}_{2,1}^{t}\right) = \tilde{\mathrm{f}}_{2,1}\left(x_7^t,x_3^t,x_4^t,x_5^t\right)$$

$$\left(V_{3,1}^{in},V_{3,1}\right) = \left(\left\{x_3\right\},\left\{x_1,x_2\right\}\right), \qquad \mathbb{X}_{3,1}^{t+1} = \tilde{\mathrm{f}}_{3,1}\left(\mathbb{X}_{3,1}^{in,t},\mathbb{X}_{3,1}^{t}\right) = \tilde{\mathrm{f}}_{3,1}\left(x_3^t,x_1^t,x_2^t\right)$$

$$\left(V_{4,1}^{in},V_{4,1}\right) = \left(\left\{x_1,x_3,x_6\right\},\left\{x_{10},x_{11}\right\}\right), \quad \mathbb{X}_{4,1}^{t+1} = \tilde{\mathrm{f}}_{4,1}\left(\mathbb{X}_{4,1}^{in,t},\mathbb{X}_{4,1}^{t}\right) = \tilde{\mathrm{f}}_{4,1}\left(x_1^t,x_3^t,x_6^t,x_{10}^t,x_{11}^t\right)$$

and $V_{4,1}^{out} = \varnothing$.

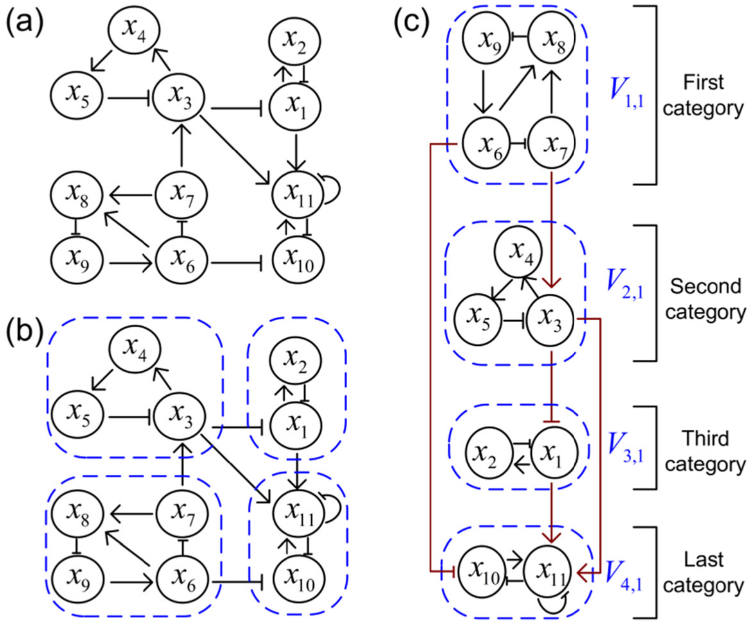

S.Fig. 1. Hierarchical partition

Now we describe the way to construct attractors of the given network based on the hierarchical partition.

S.Definition 2. The definition of attractors of the $(1,k)$ subnetworks $V_{1,k}$ is the same as that of the whole network since there are no input nodes to $V_{1,k}$. Attractors of $V_{1,k}$ are also called "local attractors" of $V_{1,k}$ or simply "attractors of $V_{1,k}$". The set of attractors of $V_{1,k}$ is denoted by

$$A_{1,k}^{loc}=\left\{\mathbb{a}_{\langle :1,k\rangle}^{1},\cdots,\mathbb{a}_{\langle :1,k\rangle}^{\eta_{1,k}}\right\}.$$

Let $V_1=V_{1,1}\cup\cdots\cup V_{1,\tau_1}$ and the set of attractors of V_1 be written as

$$A_1^{loc}=A_{1,1}^{loc}\oplus\cdots\oplus A_{1,\tau_1}^{loc}.$$

Due to the condition C1 above, the set of attractors of V_1 becomes

$$\begin{aligned}A_1^{loc}&=A_{1,1}^{loc}\oplus\cdots\oplus A_{1,\tau_1}^{loc}\\&=\bigcup_{(k_1,\cdots,k_{\tau_1})\in\prod_{j=1}^{\tau_1}\{1,\cdots,\eta_{1,j}\}}\left(\left(\left(\mathbb{a}_{\langle :1,1\rangle}^{k_1}\oplus\mathbb{a}_{\langle :1,2\rangle}^{k_2}\right)\oplus\mathbb{a}_{\langle :1,3\rangle}^{k_3}\right)\oplus\cdots\right)\oplus\mathbb{a}_{\langle :1,\tau_1\rangle}^{k_{\tau_1}}\\&=\left\{\mathbb{a}_{\langle 1\rangle},\cdots,\mathbb{a}_{\langle\omega_1\rangle}\right\},\end{aligned}$$

Here for two attractors $\mathbb{a}=[\![a^0,\cdots,a^{n_1-1}]\!]\in A_{1,i}^{loc}$ and $\mathbb{b}=[\![b^0,\cdots,b^{n_2-1}]\!]\in A_{1,j}^{loc}\ (i\neq j, n_1\leq n_2)$, we define

$$\mathbb{a}\oplus\mathbb{b}=\left\{[\![a^k b^0]\!]\,\middle|\,0\leq k\leq n_1-1\right\}$$

and $[a_k b_1]$ denotes the attractor $[\![a^k b^0, a^{k+1}b^1,\cdots,a^{k+\mathrm{lcm}(n_1,n_2)-1}b^{\mathrm{lcm}(n_1,n_2)-1}]\!]$ with the least common multiplier $\mathrm{lcm}(n_1,n_2)$ of n_1 and n_2. The operator $A\oplus B$ for the two sets of attractors A and B is also defined as

$$A \oplus B = \left\{ \mathbb{a}_i \oplus \mathbb{b}_j \middle| 1 \le i \le n_3, 1 \le j \le n_4 \right\}$$

for $A = \{\mathbb{a}_1, \cdots, \mathbb{a}_{n_3}\}$ and $B = \{\mathbb{b}_1, \cdots, \mathbb{b}_{n_4}\}$.

For example, taking $\mathbb{a} = [\![10,01]\!] \in A_{1,1}^{loc}$ and $\mathbb{b} = [\![100,001,010]\!] \in A_{1,2}^{loc}$, we have

$$\mathbb{a} \oplus \mathbb{b} = \{[10100],[01100]\},$$
$$[10100] = [\![10100,01001,10010,01100,10001,01010]\!],$$
$$[01100] = [\![01100,10001,01010,10100,01001,10010]\!].$$

S.Example 2. In the case of S.Fig. 2, there exists only one subnetwork $V_{1,1}$ in the 1st category. Using the update rules in Additional file 7(a), we have

$$A_{1,1}^{loc} = \left\{\mathbb{a}_{\langle:1,1\rangle}^1, \mathbb{a}_{\langle:1,1\rangle}^2, \mathbb{a}_{\langle:1,1\rangle}^3\right\}, \ \mathbb{a}_{\langle:1,1\rangle}^1 = [\![10,01]\!], \mathbb{a}_{\langle:1,1\rangle}^2 = [\![00]\!], \mathbb{a}_{\langle:1,1\rangle}^3 = [\![11]\!].$$

in Additional file 7(b) and

$$V_1 = V_{1,1},\ A_1^{loc} = A_{1,1}^{loc} = \{\mathbb{a}_{<1>}, \mathbb{a}_{<2>}, \mathbb{a}_{<3>}\} = \{[\![10,01]\!],[\![00]\!],[\![11]\!]\}.$$

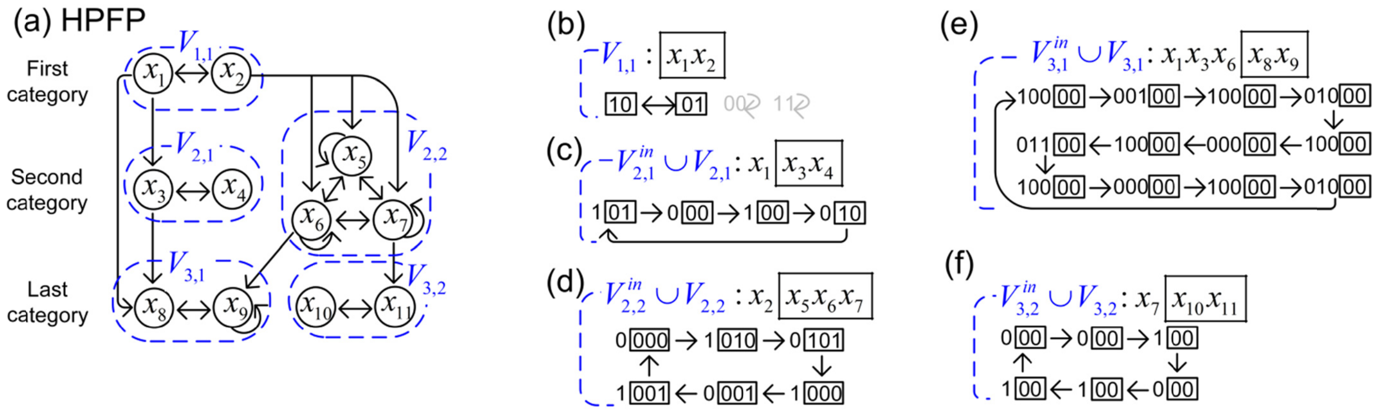

(g) Local attractors with the signal $[\![10,01]\!]$ in $V_{1,1}$

Order	x1x2	x3x4	x5x6x7	x8x9	x10x11
1	10	01	000	00	00
2	01	00	010		
3		00	101		
4		10	000		
5			001		
6			001		

(h) Global attractor of (a) with the signal $[\![10,01]\!]$ in $V_{1,1}$

x1x2	x3x4	x5x6x7	x8x9	x10x11
10	01	000	00	00
01	00	010	00	00
10	00	101	00	00
01	10	000	00	00
10	01	001	00	00
01	00	001	00	00
10	00	000	00	00
01	10	010	00	00
10	01	101	00	00
01	00	000	00	00
10	00	001	00	00
01	10	001	00	00

S.Fig. 2. Process for sequential concatenation of local attractors to construct the global attractors

Differently from the subnetworks in the first category, the $(2,k)$ subnetworks in the second category have input nodes and then we need to consider the input signals for definition of attractors of the $(2,k)$ subnetworks.

S.Definition 3. Assume that $V_{2,k}^{in}=\left\{x_{(2,k),1}^{in},\cdots,x_{(2,k),\rho_{(2,k)}}^{in}\right\}$ is nonempty and consider a cyclic attractor $\mathbb{a}_{\langle j\rangle}$ of length p in the first category. Then the symbol $\mathbb{a}_{\langle j\rangle to(2,k)}^{t}\left(0\le t\le p-1\right)$ denotes the states of the nodes $x_{(2,k),\ell}^{in}\left(1\le \ell\le \rho_{(2,k)}\right)$ obtained from $\mathbb{a}_{\langle j\rangle}^{t}$.

For example, taking $V_{2,1}^{in}=\{x_1\}$ and $\mathbb{a}_{\langle 1\rangle}=[\![10,01]\!]$ of period 2 in S.Example 1, we have $\mathbb{a}_{\langle 1\rangle to(2,1)}^{0}$ denotes the states of the node x_1 obtained from $\mathbb{a}_{\langle 1\rangle}^{0}=10$, which gives $\mathbb{a}_{\langle 1\rangle to(2,1)}^{0}=0$. Similarly, we have $\mathbb{a}_{\langle 1\rangle to(2,1)}^{1}=1$.

S.Definition 4. Two attractors $\mathbb{a}$ and $\mathbb{b}$ of a Boolean network are equal in its state transition graph if there exists t_0 such that

$$\mathbb{a}^{t+t_0}=\mathbb{b}^{t} \text{ for all } t.$$

For example, two attractors $\mathbb{a}=[\![10,01]\!]$ and $\mathbb{b}=[\![01,10]\!]$ for nodes (x_1,x_2) are equal since $\mathbb{a}^{t+1}=\mathbb{b}^{t}$ for all t.

S.Theorem 1. Let $\bigcup_{i=1}^{m}\left\{V_{i,1},\cdots,V_{i,\tau_i}\right\}$ be a hierarchical partition of V.

For an attractor $\mathbb{a}_{<j>}\in A_1^{loc}$ of period p, denote

$$\left(a^0,a^1,\cdots,a^{p-1}\right)=\left(\mathbb{a}_{\langle j\rangle to(2,k)}^{0},\cdots,\mathbb{a}_{\langle j\rangle to(2,k)}^{p-1}\right).$$

Let $A_{2,k}^{loc}\left(\mathbb{a}_{\langle j\rangle},a^0\right)$ denote the set of attractors of $V_{2,k}$ with the update rules

$$x_{(2,k),j}^{t+1}=\tilde{f}_{(2,k),j}\left(a^t,\mathbb{x}_{(2,k)}^{t}\right),\ t=0,1,\cdots,j=1,\cdots,\theta_{(2,k)}.$$

Then for $b^t=a^{t+1}\left(t=0,1,\cdots\right)$

$$A_{2,k}^{loc}\left(\mathbb{a}_{\langle j\rangle},a^0\right)=A_{2,k}^{loc}\left(\mathbb{a}_{\langle j\rangle},b^0\right).$$

Proof.

We first show that

$$A_{2,k}^{loc}\left(\mathbb{a}_{\langle j\rangle},a^0\right)\subset A_{2,k}^{loc}\left(\mathbb{a}_{\langle j\rangle},b^0\right).$$

Let $[\![\Lambda_{(2,k)}^{0},\cdots,\Lambda_{(2,k)}^{\ell p-1}]\!]\in A_{2,k}^{loc}\left(\mathbb{a}_{\langle j\rangle},a^0\right)$ for some $\ell\ge 1$, which means that

$$\Lambda_{(2,k)}^{t}\left(0\le t\le \ell p-1\right) \text{ are pairwise distinct,}$$

and

$$\left(\tilde{\mathbb{f}}_{2,k}\left(a^0,\Lambda_{(2,k)}^{0}\right),\cdots,\tilde{\mathbb{f}}_{2,k}\left(a^{\ell p-2},\Lambda_{(2,k)}^{\ell p-2}\right),\tilde{\mathbb{f}}_{2,k}\left(a^{\ell p-1},\Lambda_{(2,k)}^{\ell p-1}\right)\right)=\left(\Lambda_{(2,k)}^{1},\cdots,\Lambda_{(2,k)}^{\ell p-1},\Lambda_{(2,k)}^{0}\right).$$

Since

$$\Lambda_{(2,k)}^{t+1}\left(0\le t\le \ell p-1\right) \text{ are pairwise distinct,}$$

and

$$\left(\tilde{f}_{2,k}\left(b^0,\Lambda^1_{(2,k)}\right),\cdots,\tilde{f}_{2,k}\left(b^{\ell p-2},\Lambda^{\ell p-1}_{(2,k)}\right),\tilde{f}_{2,k}\left(b^{\ell p-1},\Lambda^{\ell p}_{(2,k)}\right)\right)$$
$$=\left(\tilde{f}_{2,k}\left(a^1,\Lambda^1_{(2,k)}\right),\cdots,\tilde{f}_{2,k}\left(a^{\ell p-1},\Lambda^{\ell p-1}_{(2,k)}\right),\tilde{f}_{2,k}\left(a^0,\Lambda^0_{(2,k)}\right)\right)$$
$$=\left(\Lambda^2_{(2,k)},\cdots,\Lambda^{\ell p-1}_{(2,k)},\Lambda^0_{(2,k)},\Lambda^1_{(2,k)}\right),$$

which implies

$$\llbracket \Lambda^1_{(2,k)},\cdots,\Lambda^{\ell p-1}_{(2,k)},\Lambda^0_{(2,k)} \rrbracket \in A^{loc}_{2,k}\left(\mathbb{a}_{\langle j\rangle},b^0\right).$$

Therefore

$$\llbracket \Lambda^0_{(2,k)},\cdots,\Lambda^{\ell p-1}_{(2,k)} \rrbracket \in A^{loc}_{2,k}\left(\mathbb{a}_{\langle j\rangle},b^0\right),$$

so that we have $A^{loc}_{2,k}\left(\mathbb{a}_{\langle j\rangle},a^0\right)\subset A^{loc}_{2,k}\left(\mathbb{a}_{\langle j\rangle},b^0\right)$.

Similarly we can have $A^{loc}_{2,k}\left(\mathbb{a}_{\langle j\rangle},a^0\right)\supset A^{loc}_{2,k}\left(\mathbb{a}_{\langle j\rangle},b^0\right)$, which completes the proof.

S. Example 3. (1) For $\mathbb{a}_{\langle 1\rangle}=\llbracket 10,01 \rrbracket$ in S.Fig. 2, we have

$$V^{in}_{2,1}=\{x_1\} \text{ and } \left(x^0_1,x^1_1\right)=\left(a^0,a^1\right)=(1,0)$$

and then in Additional file 7(c)

t=0			t=1			t=2			t=3			t=4		
x1	x3	x4	x1	x3	x4	x1	x3	x4	x1	x3	x4	x1	x3	x4
1	1	1	0	0	1	1	0	0	0	1	0	1	0	1
1	1	0	0	1	1	1	0	1	0	0	0	1	0	0
1	0	1	**0**	**0**	**0**	**1**	**0**	**0**	**0**	**1**	**0**	**1**	**0**	**1**
1	0	0	0	1	0	1	0	1	0	0	0	1	0	0

which gives

$$A^{loc}_{2,1}\left(\mathbb{a}_{\langle 1\rangle},a^0\right)=\left\{\llbracket 01,00,00,10 \rrbracket\right\}$$

Similarly, letting $\left(x^0_1,x^1_1\right)=\left(b^0,b^1\right)=(0,1)$, we have in Additional file 7(c)

t=0			t=1			t=2			t=3			t=4		
x1	x3	x4	x1	x3	x4	x1	x3	x4	x1	x3	x4	x1	x3	x4
0	1	1	1	0	1	0	0	0	1	0	0	0	1	0
0	1	0	1	0	1	0	0	0	1	0	0	0	1	0
0	0	1	**1**	**0**	**0**	**0**	**1**	**0**	**1**	**0**	**1**	**0**	**0**	**0**
0	0	0	1	0	0	0	1	0	1	0	1	0	0	0

and then

$$A^{loc}_{2,1}\left(\mathbb{a}_{\langle 1\rangle},b^0\right)=\left\{\llbracket 00,10,01,00 \rrbracket\right\}.$$

Therefore

$$A^{loc}_{2,1}\left(\mathbb{a}_{\langle 1\rangle},a^0\right)=A^{loc}_{2,1}\left(\mathbb{a}_{\langle 1\rangle},b^0\right).$$

(2) For fixed attractors $\mathbb{a}_{\langle 2\rangle}=\llbracket 00 \rrbracket$ and $\mathbb{a}_{\langle 3\rangle}=\llbracket 11 \rrbracket$ in S.Fig. 2, we have $a^0=b^0$ and then

$$A_{2,1}^{loc}\left(\mathbb{a}_{\langle 2\rangle},a^0\right)=A_{2,1}^{loc}\left(\mathbb{a}_{\langle 2\rangle},b^0\right)=\left\{\llbracket 00\rrbracket\right\},$$
$$A_{2,1}^{loc}\left(\mathbb{a}_{\langle 3\rangle},a^0\right)=A_{2,1}^{loc}\left(\mathbb{a}_{\langle 3\rangle},b^0\right)=\left\{\llbracket 00,10,11,01\rrbracket\right\}.$$

S.Remark 1. S.Theorem 1 implies that the starting state value of input signal from attractors to the subnetwork $V_{i,k}$ with preserving the order of the input signal does not change the set of local attractors of $V_{i,k}$. Finally we can define attractors of $V_{2,k}$.

S.Definition 5. ($V_{2,k}\left(\mathbb{a}_{\langle j\rangle}\right)$, $A_{2,k}^{par}\left(\mathbb{a}_{\langle j\rangle}\right)$, $A_{2,k}^{loc}\left(\mathbb{a}_{\langle j\rangle}\right)$ For an attractor $\mathbb{a}_{\langle j\rangle}\in A_1^{loc}$, define

$$V_{2,k}\left(\mathbb{a}_{\langle j\rangle}\right)=\left\{\mathbb{a}_{\langle j\rangle to(2,k)}\right\}\cup V_{2,k}.$$

The symbol $\left\llbracket \mathbb{a}^0_{\langle j\rangle to(2,k)}\Lambda^0_{(2,k)},\cdots,\mathbb{a}^{\tau p-1}_{\langle j\rangle to(2,k)}\Lambda^{\tau p-1}_{(2,k)}\right\rrbracket$ for some positive integer τ denotes an attractor of $V_{2,k}\left(\mathbb{a}_{\langle j\rangle}\right)$ with length τp, which means that

$$\mathbb{a}^t_{\langle j\rangle to(2,k)}\Lambda^t_{(2,k)}\left(0\le t\le \tau p-1\right) \text{ are pairwise distinct,}$$
$$\left(\tilde{\mathrm{f}}_{2,k}\left(\mathbb{a}^0_{\langle j\rangle to(2,k)},\Lambda^0_{(2,k)}\right),\cdots,\tilde{\mathrm{f}}_{2,k}\left(\mathbb{a}^{\tau p-2}_{\langle j\rangle to(2,k)},\Lambda^{\tau p-2}_{(2,k)}\right),\tilde{\mathrm{f}}_{2,k}\left(\mathbb{a}^{\tau p-1}_{\langle j\rangle to(2,k)},\Lambda^{\tau p-1}_{(2,k)}\right)\right)=\left(\Lambda^1_{(2,k)},\cdots,\Lambda^{\tau p-1}_{(2,k)},\Lambda^0_{(2,k)}\right).$$

Here the attractor is also called "a partial attractor" of $V_{2,k}\left(\mathbb{a}_{\langle j\rangle}\right)$.

In this case, we can obtain the periodic sequence $\left\llbracket \Lambda^0_{(2,k)},\cdots,\Lambda^{q-1}_{(2,k)}\right\rrbracket$ of $V_{2,k}\left(\mathbb{a}_{\langle j\rangle}\right)$ with period q which is one divisor of τp. The periodic sequence is called "a local attractor" of $V_{2,k}\left(\mathbb{a}_{\langle j\rangle}\right)$.

The sets of partial attractors and local attractors of $V_{2,k}\left(\mathbb{a}_{\langle j\rangle}\right)$ are denoted by $A_{2,k}^{par}\left(\mathbb{a}_{\langle j\rangle}\right)$ and $A_{2,k}^{loc}\left(\mathbb{a}_{\langle j\rangle}\right)$, respectively.

S.Example 4. For $\mathbb{a}_{<1>}=\llbracket 10,01\rrbracket$, $\mathbb{a}_{\langle 2\rangle}=\llbracket 00\rrbracket$ and $\mathbb{a}_{<3>}=\llbracket 11\rrbracket$ in S.Fig. 2, we have in Additional file 7(d)

$$V_{2,1}\left(\mathbb{a}_{<1>}\right)=\left\{1,0,x_3,x_4\right\},\quad A_{2,1}^{par}\left(\mathbb{a}_{<1>}\right)=\left\{\llbracket 101,000,100,010\rrbracket\right\},\quad A_{2,1}^{loc}\left(\mathbb{a}_{<1>}\right)=\left\{\llbracket 01,00,00,10\rrbracket\right\},$$
$$V_{2,2}\left(\mathbb{a}_{<1>}\right)=\left\{0,1,x_5,x_6,x_7\right\},\quad A_{2,2}^{par}\left(\mathbb{a}_{<1>}\right)=\left\{\llbracket 0000,1010,0101,1000,0001,1001\rrbracket\right\},$$
$$A_{2,2}^{loc}\left(\mathbb{a}_{<1>}\right)=\left\{\llbracket 000,010,101,000,001,001\rrbracket\right\},$$
$$V_{2,1}\left(\mathbb{a}_{<2>}\right)=\left\{0,x_3,x_4\right\},\quad A_{2,1}^{par}\left(\mathbb{a}_{<2>}\right)=\left\{\llbracket 000\rrbracket\right\},\quad A_{2,1}^{loc}\left(\mathbb{a}_{<2>}\right)=\left\{\llbracket 00\rrbracket\right\},$$
$$V_{2,2}\left(\mathbb{a}_{<2>}\right)=\left\{0,x_5,x_6,x_7\right\},\quad A_{2,2}^{par}\left(\mathbb{a}_{<2>}\right)=\left\{\llbracket 0000,1010,0101,1000,0001,1001\rrbracket\right\},$$
$$A_{2,2}^{loc}\left(\mathbb{a}_{<2>}\right)=\left\{\llbracket 000,010,101,000,001,001\rrbracket\right\}$$

and

$$V_{2,1}\left(\mathbb{a}_{<3>}\right)=\left\{1,x_3,x_4\right\},\quad A_{2,1}^{par}\left(\mathbb{a}_{<3>}\right)=\left\{\llbracket 101,110,111,101\rrbracket\right\},\quad A_{2,1}^{loc}\left(\mathbb{a}_{<3>}\right)=\left\{\llbracket 01,10,11,01\rrbracket\right\},$$
$$V_{2,2}\left(\mathbb{a}_{<3>}\right)=\left\{1,x_5,x_6,x_7\right\},\quad A_{2,2}^{par}\left(\mathbb{a}_{<3>}\right)=\left\{\llbracket 11100\rrbracket,\llbracket 11001,11000\rrbracket\right\},$$
$$A_{2,2}^{loc}\left(\mathbb{a}_{<3>}\right)=\left\{\llbracket 100\rrbracket,\llbracket 001,000\rrbracket\right\}.$$

S.Definition 6. The symbol $\llbracket \mathbb{a}^{0}_{\langle j_1,\cdots,j_{i-1}\rangle}\Lambda^{0}_{(i,k)},\cdots,\mathbb{a}^{p-1}_{\langle j_1,\cdots,j_{i-1}\rangle}\Lambda^{p-1}_{(i,k)} \rrbracket$ is called "a concatenated attractor" of $V_{i,k}\left(\mathbb{a}_{\langle j_1,\cdots,j_{i-1}\rangle}\right)$ for $2 \le i \le m$. The set of concatenated attractors of $V_{i,k}\left(\mathbb{a}_{\langle j_1,\cdots,j_{i-1}\rangle}\right)$ is denoted by $A^{con}_{i,k}\left(\mathbb{a}_{\langle j_1,\cdots,j_{i-1}\rangle}\right)$ with

$$A^{con}_{i,k}\left(\mathbb{a}_{\langle j_1,\cdots,j_{i-1}\rangle}\right)=\left\{\mathbb{a}^{1}_{\langle j_1,\cdots,j_{i-1}:i,k\rangle},\cdots,\mathbb{a}^{\eta_{i,k}(j_1,\cdots,j_{i-1})}_{\langle j_1,\cdots,j_{i-1}:i,k\rangle}\right\},\ 1 \le k \le \omega_i .$$

Three kinds of attractors of $V_{i,k}\left(\mathbb{a}_{\langle j_1,\cdots,j_{i-1}\rangle}\right)$ have been defined: concatenated, partial and local attractors, which are elements of the sets $A^{con}_{i,k}\left(\mathbb{a}_{\langle j_1,\cdots,j_{i-1}\rangle}\right)$, $A^{par}_{i,k}\left(\mathbb{a}_{\langle j_1,\cdots,j_{i-1}\rangle}\right)$ and $A^{loc}_{i,k}\left(\mathbb{a}_{\langle j_1,\cdots,j_{i-1}\rangle}\right)$, respectively.

Let

$$V_i\left(\mathbb{a}_{\langle j_1,\cdots,j_{i-1}\rangle}\right)=V_{i,1}\left(\mathbb{a}_{\langle j_1,\cdots,j_{i-1}\rangle}\right)\cup\cdots\cup V_{i,\tau_i}\left(\mathbb{a}_{\langle j_1,\cdots,j_{i-1}\rangle}\right)$$

and

$$A^{con}_{i}\left(\mathbb{a}_{\langle j_1,\cdots,j_{i-1}\rangle}\right)=A^{con}_{i,1}\left(\mathbb{a}_{\langle j_1,\cdots,j_{i-1}\rangle}\right)\dot{+}\cdots\dot{+}A^{con}_{i,\tau_i}\left(\mathbb{a}_{\langle j_1,\cdots,j_{i-1}\rangle}\right)$$

be the set of concatenated attractors of $V_i\left(\mathbb{a}_{\langle j_1,\cdots,j_{i-1}\rangle}\right)$.

Then

$$\begin{aligned}A^{con}_{i}\left(\mathbb{a}_{\langle j_1,\cdots,j_{i-1}\rangle}\right)&=A^{con}_{i,1}\left(\mathbb{a}_{\langle j_1,\cdots,j_{i-1}\rangle}\right)\dot{+}\cdots\dot{+}A^{con}_{i,\tau_i}\left(\mathbb{a}_{\langle j_1,\cdots,j_{i-1}\rangle}\right)\\&=\bigcup_{(k_1,\cdots,k_{\tau_1})\in\prod_{j=1}^{\tau_i}\{1,\cdots,\eta_{i,j}(\omega_{i-1})\}}\left(\left(\left(\mathbb{a}^{k_1}_{\langle j_1,\cdots,j_{i-1}:i,1\rangle}\dot{+}\mathbb{a}^{k_2}_{\langle j_1,\cdots,j_{i-1}:i,2\rangle}\right)\dot{+}\mathbb{a}^{k_3}_{\langle j_1,\cdots,j_{i-1}:i,3\rangle}\right)\dot{+}\cdots\right)\dot{+}\mathbb{a}^{k_{\tau_i}}_{\langle j_1,\cdots,j_{i-1}:i,\tau_i\rangle}\\&=\left\{\mathbb{a}_{\langle j_1,\cdots,j_{i-1},1\rangle},\cdots,\mathbb{a}_{\langle j_1,\cdots,j_{i-1},\omega_i\rangle}\right\}\end{aligned}$$

where the operator $\dot{+}$ is defined as

$$\mathbb{b}\dot{+}\mathbb{c}=\left\llbracket \mathbb{a}^{0}_{\langle j_1,\cdots,j_{i-1}\rangle}x^0y^0,\mathbb{a}^{1}_{\langle j_1,\cdots,j_{i-1}\rangle}x^1y^1,\cdots,\mathbb{a}^{\mathrm{lcm}(p,q)-1}_{\langle j_1,\cdots,j_{i-1}\rangle}x^{\mathrm{lcm}(p,q)-1}y^{\mathrm{lcm}(p,q)-1}\right\rrbracket$$

for $\mathbb{b}=\left\llbracket \mathbb{a}^{0}_{\langle j_1,\cdots,j_{i-1}\rangle}x^0,\cdots,\mathbb{a}^{p-1}_{\langle j_1,\cdots,j_{i-1}\rangle}x^{p-1}\right\rrbracket$ and $\mathbb{c}=\left\llbracket \mathbb{a}^{0}_{\langle j_1,\cdots,j_{i-1}\rangle}y^0,\cdots,\mathbb{a}^{q-1}_{\langle j_1,\cdots,j_{i-1}\rangle}y^{q-1}\right\rrbracket$.

S.Example 5. For $\mathbb{a}_{\langle 1\rangle}=\llbracket 10,01\rrbracket$ in S.Fig. 2, we have

$$A^{con}_{2,1}\left(\mathbb{a}_{\langle 1\rangle}\right)=\left\{\llbracket 1001,0100,1000,0110\rrbracket\right\}=\left\{\mathbb{a}^{1}_{\langle 1:2,1\rangle}\right\}.$$

Similarly, we have

$$A^{con}_{2,2}\left(\mathbb{a}_{\langle 1\rangle}\right)=\left\{\llbracket 10000,01010,10101,01000,10001,01001\rrbracket\right\}=\left\{\mathbb{a}^{1}_{\langle 1:2,2\rangle}\right\}.$$

Therefore

$$A_2^{con}\left(\mathbb{a}_{\langle 1\rangle}\right) = A_{2,1}^{con}\left(\mathbb{a}_{\langle 1\rangle}\right) \dot{+} A_{2,2}^{con}\left(\mathbb{a}_{\langle 1\rangle}\right) = \left\{\mathbb{a}^1_{\langle 1:2,1\rangle}\right\} \dot{+} \left\{\mathbb{a}^1_{\langle 1:2,2\rangle}\right\} = \mathbb{a}^1_{\langle 1:2,1\rangle} \dot{+} \mathbb{a}^1_{\langle 1:2,2\rangle} = \{[1001000]\}$$

$$= \left\{\left\llbracket \begin{matrix} 1001000, 0100010, 1000101, 0110000, 1001001, 0100001, \\ 1000000, 0110010, 1001101, 0100000, 1000001, 0110001 \end{matrix} \right\rrbracket\right\}$$

$$= \left\{\mathbb{a}_{\langle 1,1\rangle}\right\}.$$

Here the symbol $\left\llbracket \begin{matrix} 1001000, 0100010, 1000101, 0110000, 1001001, 0100001, \\ 1000000, 0110010, 1001101, 0100000, 1000001, 0110001 \end{matrix} \right\rrbracket$ denotes the state transition

$$\to 1001000 \to 0100010 \to 1000101 \to 0110000 \to 1001001 \to 0100001$$

$$1000000 \to 0110010 \to 1001101 \to 0100000 \to 1000001 \to 0110001$$

S.Example 6. For $\mathbb{a}_{\langle 2\rangle} = \llbracket 00 \rrbracket$ and $\mathbb{a}_{\langle 3\rangle} = \llbracket 11 \rrbracket$ in S.Fig. 2,

$$A_{2,1}^{con}\left(\mathbb{a}_{\langle 2\rangle}\right) = \{\llbracket 0000 \rrbracket\} = \left\{\mathbb{a}^1_{\langle 2:2,1\rangle}\right\},$$

$$A_{2,2}^{con}\left(\mathbb{a}_{\langle 2\rangle}\right) = \{\llbracket 00010, 00110, 00100 \rrbracket, \llbracket 00001 \rrbracket\} = \left\{\mathbb{a}^1_{\langle 2:2,2\rangle}, \mathbb{a}^2_{\langle 2:2,2\rangle}\right\}$$

and

$$A_{2,1}^{con}\left(\mathbb{a}_{<3>}\right) = \{\llbracket 1100, 1110, 1111, 1101 \rrbracket\} = \left\{\mathbb{a}^1_{\langle 3:2,1\rangle}\right\},$$

$$A_{2,2}^{con}\left(\mathbb{a}_{<3>}\right) = \{\llbracket 11100 \rrbracket, \llbracket 11001, 11000 \rrbracket\} = \left\{\mathbb{a}^1_{\langle 3:2,2\rangle}, \mathbb{a}^2_{\langle 3:2,2\rangle}\right\}.$$

Therefore

$$A_2^{con}\left(\mathbb{a}_{<2>}\right) = A_{2,1}^{con}\left(\mathbb{a}_{<2>}\right) \dot{+} A_{2,2}^{con}\left(\mathbb{a}_{<2>}\right) = \left\{\mathbb{a}^1_{\langle 2:2,1\rangle}\right\} \dot{+} \left\{\mathbb{a}^1_{\langle 2:2,2\rangle}, \mathbb{a}^2_{\langle 2:2,2\rangle}\right\}$$

$$= \left(\mathbb{a}^1_{\langle 2:2,1\rangle} \dot{+} \mathbb{a}^1_{\langle 2:2,2\rangle}\right) \cup \left(\mathbb{a}^1_{\langle 2:2,1\rangle} \dot{+} \mathbb{a}^2_{\langle 2:2,2\rangle}\right) = \{[0000010]\} \cup \{[0000001]\}$$

$$= \{\llbracket 0000010, 0000110, 0000100 \rrbracket\} \cup \{\llbracket 0000001 \rrbracket\}$$

$$= \left\{\mathbb{a}_{\langle 2,1\rangle}, \mathbb{a}_{\langle 2,2\rangle}\right\}$$

and

$$A_2^{con}\left(\mathbb{a}_{<3>}\right) = A_{2,1}^{con}\left(\mathbb{a}_{<3>}\right) \dot{+} A_{2,2}^{con}\left(\mathbb{a}_{<3>}\right) = \left\{\mathbb{a}^1_{\langle 3:2,1\rangle}\right\} \dot{+} \left\{\mathbb{a}^1_{\langle 3:2,2\rangle}, \mathbb{a}^2_{\langle 3:2,2\rangle}\right\}$$

$$= \left(\mathbb{a}^1_{\langle 3:2,1\rangle} \dot{+} \mathbb{a}^1_{\langle 3:2,2\rangle}\right) \cup \left(\mathbb{a}^1_{\langle 3:2,1\rangle} \dot{+} \mathbb{a}^2_{\langle 3:2,2\rangle}\right) = \{[1100100]\} \cup \{[1100001], [1100000]\}$$

$$= \{\llbracket 1100100, 1110100, 1111100, 1101100 \rrbracket\} \cup \left\{\left\llbracket \begin{matrix} 1100001, 1110000, \\ 1111001, 1101000 \end{matrix} \right\rrbracket, \left\llbracket \begin{matrix} 1100000, 1110001, \\ 1111000, 1101001 \end{matrix} \right\rrbracket\right\}$$

$$= \left\{\mathbb{a}_{\langle 3,1\rangle}, \mathbb{a}_{\langle 3,2\rangle}, \mathbb{a}_{\langle 3,3\rangle}\right\}$$

S.Definition 7. The set of concatenated attractors of $V_i = \bigcup_{(j_1,\cdots,j_{i-1}) \in \prod_{\ell=1}^{i-1} \omega_\ell} V_i\left(\mathbb{a}_{\langle j_1,\cdots,j_{i-1}\rangle}\right)$ for $2 \le i \le m$ is denoted

by

$$A_i^{con} = \bigcup_{(j_1,\cdots,j_{i-1})\in\prod_{\ell=1}^{i-1}\omega_\ell} A_i^{con}\left(\mathbb{a}_{\langle j_1,\cdots,j_{i-1}\rangle}\right).$$

S.Example 7. From S.Fig. 2, we have

$$\begin{aligned}
A_2^{con} &= A_2^{con}\left(\mathbb{a}_{\langle 1\rangle}\right) \cup A_2^{con}\left(\mathbb{a}_{\langle 2\rangle}\right) \cup A_2^{con}\left(\mathbb{a}_{\langle 3\rangle}\right) \\
&= \left\{\mathbb{a}_{\langle 1,1\rangle}\right\} \cup \left\{\mathbb{a}_{\langle 2,1\rangle}, \mathbb{a}_{\langle 2,2\rangle}\right\} \cup \left\{\mathbb{a}_{\langle 3,1\rangle}, \mathbb{a}_{\langle 3,2\rangle}, \mathbb{a}_{\langle 3,3\rangle}\right\} \\
&= \left\{\begin{array}{l}
\left\llbracket \begin{array}{l} 1001000, 0100010, 1000101, 0110000, 1001001, 0100001, \\ 1000000, 0110010, 1001101, 0100000, 1000001, 0110001 \end{array} \right\rrbracket, \\
\llbracket 0000010, 0000110, 0000100 \rrbracket, \llbracket 0000001 \rrbracket \\
\left\llbracket \begin{array}{l} 1100100, 1110100, \\ 1111100, 1101100 \end{array} \right\rrbracket, \left\llbracket \begin{array}{l} 1100001, 1110000, \\ 1111001, 1101000 \end{array} \right\rrbracket, \left\llbracket \begin{array}{l} 1100000, 1110001, \\ 1111000, 1101001 \end{array} \right\rrbracket
\end{array}\right\}
\end{aligned}$$

S.Example 8. For $\mathbb{a}_{\langle 1,1\rangle} = \left\llbracket \begin{array}{l} 1001000, 0100010, 1000101, 0110000, 1001001, 0100001, \\ 1000000, 0110010, 1001101, 0100000, 1000001, 0110001 \end{array} \right\rrbracket$ in S.Fig. 2, we have in Additional files 7(e) and 7(f)

$$A_{3,1}^{con}\left(\mathbb{a}_{\langle 1,1\rangle}\right) = \left\{\left\llbracket \begin{array}{l} 100100000, 010001000, 100010100, 011000000, 100100100, 010000100, \\ 100000000, 011001000, 100110100, 010000000, 100000100, 011000100 \end{array} \right\rrbracket\right\} = \left\{\mathbb{a}^1_{\langle 1,1:3,1\rangle}\right\},$$

$$A_{3,2}^{con}\left(\mathbb{a}_{\langle 1,1\rangle}\right) = \left\{\left\llbracket \begin{array}{l} 100100000, 010001000, 100010100, 011000000, 100100100, 010000100, \\ 100000000, 011001000, 100110100, 010000000, 100000100, 011000100 \end{array} \right\rrbracket\right\} = \left\{\mathbb{a}^1_{\langle 1,1:3,2\rangle}\right\}.$$

Therefore

$$\begin{aligned}
A_3^{con}\left(\mathbb{a}_{\langle 1,1\rangle}\right) &= A_{3,1}^{con}\left(\mathbb{a}_{\langle 1,1\rangle}\right) \dotplus A_{3,2}^{con}\left(\mathbb{a}_{\langle 1,1\rangle}\right) = \left\{\mathbb{a}^1_{\langle 1,1:3,1\rangle}\right\} \dotplus \left\{\mathbb{a}^1_{\langle 1,1:3,2\rangle}\right\} = \mathbb{a}^1_{\langle 1,1:3,1\rangle} \dotplus \mathbb{a}^1_{\langle 1,1:3,2\rangle} = \{[10010000000]\} \\
&= \left\{\left\llbracket \begin{array}{l} 10010000000, 01000100000, 10001010000, 01100000000, 10010010000, 01000010000, \\ 10000000000, 01100100000, 10011010000, 01000000000, 10000010000, 01100010000 \end{array} \right\rrbracket\right\} \\
&= \left\{\mathbb{a}_{\langle 1,1,1\rangle}\right\}.
\end{aligned}$$

S.Example 9. Similarly for $\mathbb{a}_{\langle 2,1\rangle} = \llbracket 0000010, 0000110, 0000100 \rrbracket$, we have

$$A_{3,1}^{con}\left(\mathbb{a}_{\langle 2,1\rangle}\right) = \left\{\llbracket 000001000, 000011000, 000010000 \rrbracket\right\} = \left\{\mathbb{a}^1_{\langle 2,1:3,1\rangle}\right\},$$

$$A_{3,2}^{con}\left(\mathbb{a}_{\langle 2,1\rangle}\right) = \left\{\llbracket 000001000, 000011000, 000010000 \rrbracket\right\} = \left\{\mathbb{a}^1_{\langle 2,1:3,2\rangle}\right\}.$$

Therefore

$$\begin{aligned}
A_3^{con}\left(\mathbb{a}_{\langle 2,1\rangle}\right) &= A_{3,1}^{con}\left(\mathbb{a}_{\langle 2,1\rangle}\right) \dotplus A_{3,2}^{con}\left(\mathbb{a}_{\langle 2,1\rangle}\right) = \left\{\mathbb{a}^1_{\langle 2,1:3,1\rangle}\right\} \dotplus \left\{\mathbb{a}^1_{\langle 2,1:3,2\rangle}\right\} = \mathbb{a}^1_{\langle 2,1:3,1\rangle} \dotplus \mathbb{a}^1_{\langle 2,1:3,2\rangle} = \{[00000100000]\} \\
&= \left\{\llbracket 00000100000, 00001100000, 00001000000 \rrbracket\right\} \\
&= \left\{\mathbb{a}_{\langle 2,1,1\rangle}\right\}.
\end{aligned}$$

S.Example 10. For $\mathfrak{a}_{\langle 2,2\rangle} = [\![0000001]\!]$, we have

$$A_{3,1}^{con}\left(\mathfrak{a}_{\langle 2,2\rangle}\right) = \{[\![000000100]\!]\} = \left\{\mathfrak{a}^{1}_{\langle 2,2:3,1\rangle}\right\},$$

$$A_{3,2}^{con}\left(\mathfrak{a}_{\langle 2,2\rangle}\right) = \{[\![000000100]\!],[\![000000111]\!],[\![000000110,000000101]\!]\}$$

$$= \left\{\mathfrak{a}^{1}_{\langle 2,2:3,2\rangle}, \mathfrak{a}^{2}_{\langle 2,2:3,2\rangle}, \mathfrak{a}^{3}_{\langle 2,2:3,2\rangle}\right\}.$$

Therefore

$$A_{3}^{con}\left(\mathfrak{a}_{\langle 2,2\rangle}\right) = A_{3,1}^{con}\left(\mathfrak{a}_{\langle 2,2\rangle}\right) \dot{+} A_{3,2}^{con}\left(\mathfrak{a}_{\langle 2,2\rangle}\right) = \left\{\mathfrak{a}^{1}_{\langle 2,2:3,1\rangle}\right\} \dot{+} \left\{\mathfrak{a}^{1}_{\langle 2,123,2\rangle}, \mathfrak{a}^{2}_{\langle 2,2:3,2\rangle}, \mathfrak{a}^{3}_{\langle 2,2:3,2\rangle}\right\}$$

$$= \left(\mathfrak{a}^{1}_{\langle 2,2:3,1\rangle} \dot{+} \mathfrak{a}^{1}_{\langle 2,2:3,2\rangle}\right) \cup \left(\mathfrak{a}^{1}_{\langle 2,2:3,1\rangle} \dot{+} \mathfrak{a}^{2}_{\langle 2,2:3,2\rangle}\right) \cup \left(\mathfrak{a}^{1}_{\langle 2,2:3,1\rangle} \dot{+} \mathfrak{a}^{3}_{\langle 2,2:3,2\rangle}\right)$$

$$= \{[00000010000]\} \cup \{[00000010011]\} \cup \{[00000010010]\}$$

$$= \{[\![00000010000]\!],[\![00000010011]\!],[\![00000010010,00000010001]\!]\}$$

$$= \left\{\mathfrak{a}_{\langle 2,2,1\rangle}, \mathfrak{a}_{\langle 2,2,2\rangle}, \mathfrak{a}_{\langle 2,2,3\rangle}\right\}.$$

S.Example 11. For $\mathfrak{a}_{\langle 3,1\rangle} = [\![1100100,1110100,1111100,1101100]\!]$, we have

$$A_{3,1}^{con}\left(\mathfrak{a}_{\langle 3,1\rangle}\right) = \{[\![110010000,111010000,111110000,110110000]\!]\} = \left\{\mathfrak{a}^{1}_{\langle 3,1:3,1\rangle}\right\},$$

$$A_{3,2}^{con}\left(\mathfrak{a}_{\langle 3,1\rangle}\right) = \{[\![110010000,111010000,111110000,110110000]\!]\} = \left\{\mathfrak{a}^{1}_{\langle 3,1:3,2\rangle}\right\}.$$

Therefore

$$A_{3}^{con}\left(\mathfrak{a}_{\langle 3,1\rangle}\right) = A_{3,1}^{con}\left(\mathfrak{a}_{\langle 3,1\rangle}\right) \dot{+} A_{3,2}^{con}\left(\mathfrak{a}_{\langle 3,1\rangle}\right) = \left\{\mathfrak{a}^{1}_{\langle 3,1:3,1\rangle}\right\} \dot{+} \left\{\mathfrak{a}^{1}_{\langle 3,1:3,2\rangle}\right\} = \mathfrak{a}^{1}_{\langle 3,1:3,1\rangle} \dot{+} \mathfrak{a}^{1}_{\langle 3,1:3,2\rangle} = \{[11001000000]\}$$

$$= \{[\![11001000000,11101000000,11111000000,11011000000]\!]\}$$

$$= \left\{\mathfrak{a}_{\langle 3,1,1\rangle}\right\}.$$

S.Example 12. For $\mathfrak{a}_{\langle 3,2\rangle} = [\![1100001,1110000,1111001,1101000]\!]$, we have

$$A_{3,1}^{con}\left(\mathfrak{a}_{\langle 3,2\rangle}\right) = \{[\![110000100,111000000,111100100,110100000]\!]\} = \left\{\mathfrak{a}^{1}_{\langle 3,2:3,1\rangle}\right\},$$

$$A_{3,2}^{con}\left(\mathfrak{a}_{\langle 3,2\rangle}\right) = \left\{\left[\!\!\left[\begin{matrix}110000100,111000000,\\111100100,110100000\end{matrix}\right]\!\!\right],\left[\!\!\left[\begin{matrix}110000110,111000001,\\111100110,110100001\end{matrix}\right]\!\!\right]\right\} = \left\{\mathfrak{a}^{1}_{\langle 3,2:3,2\rangle}, \mathfrak{a}^{2}_{\langle 3,2:3,2\rangle}\right\}.$$

Therefore

$$A_3^{con}\left(\mathbb{a}_{\langle 3,2\rangle}\right)=A_{3,1}^{con}\left(\mathbb{a}_{\langle 3,2\rangle}\right)\dot{+}A_{3,2}^{con}\left(\mathbb{a}_{\langle 3,2\rangle}\right)=\left\{\mathbb{a}^1_{\langle 3,2:3,1\rangle}\right\}\dot{+}\left\{\mathbb{a}^1_{\langle 3,2:3,2\rangle},\mathbb{a}^2_{\langle 3,2:3,2\rangle}\right\}$$
$$=\left(\mathbb{a}^1_{\langle 3,2:3,1\rangle}\dot{+}\mathbb{a}^1_{\langle 3,2:3,2\rangle}\right)\cup\left(\mathbb{a}^1_{\langle 3,2:3,1\rangle}\dot{+}\mathbb{a}^2_{\langle 3,2:3,2\rangle}\right)$$
$$=\{[11000010000]\}\cup\{[11000010010]\}$$
$$=\left\{\begin{bmatrix}11000010000,11100000000,\\11110010000,11010000000\end{bmatrix},\begin{bmatrix}11000010010,11100000001,\\11110010010,11010000001\end{bmatrix}\right\}$$
$$=\left\{\mathbb{a}_{\langle 3,2,1\rangle},\mathbb{a}_{\langle 3,2,2\rangle}\right\}.$$

S.Example 13. For $\mathbb{a}_{\langle 3,3\rangle}=[\![1100000,1110001,1111000,1101001]\!]$, we have

$$A_{3,1}^{con}\left(\mathbb{a}_{\langle 3,3\rangle}\right)=\{[\![110000000,111000100,111100000,110100100]\!]\}=\left\{\mathbb{a}^1_{\langle 3,3:3,1\rangle}\right\},$$
$$A_{3,2}^{con}\left(\mathbb{a}_{\langle 3,3\rangle}\right)=\left\{\begin{bmatrix}1100000000,1110001100,\\1111000000,1101001100\end{bmatrix},\begin{bmatrix}1100000001,1110001110,\\1111000001,1101001110\end{bmatrix}\right\}=\left\{\mathbb{a}^1_{\langle 3,3:3,2\rangle},\mathbb{a}^2_{\langle 3,3:3,2\rangle}\right\}.$$

Therefore

$$A_3^{con}\left(\mathbb{a}_{\langle 3,3\rangle}\right)=A_{3,1}^{con}\left(\mathbb{a}_{\langle 3,3\rangle}\right)\dot{+}A_{3,2}^{con}\left(\mathbb{a}_{\langle 3,3\rangle}\right)=\left\{\mathbb{a}^1_{\langle 3,3:3,1\rangle}\right\}\dot{+}\left\{\mathbb{a}^1_{\langle 3,3:3,2\rangle},\mathbb{a}^2_{\langle 3,3:3,2\rangle}\right\}$$
$$=\left(\mathbb{a}^1_{\langle 3,3:3,1\rangle}\dot{+}\mathbb{a}^1_{\langle 3,3:3,2\rangle}\right)\cup\left(\mathbb{a}^1_{\langle 3,3:3,1\rangle}\dot{+}\mathbb{a}^2_{\langle 3,3:3,2\rangle}\right)$$
$$=\{[11000010000]\}\cup\{[11000000001]\}$$
$$=\left\{\begin{bmatrix}11000000000,11100010000,\\11110000000,11010010000\end{bmatrix},\begin{bmatrix}11000000001,11100010010,\\11110000001,11010010010\end{bmatrix}\right\}=\left\{\mathbb{a}_{\langle 3,3,1\rangle},\mathbb{a}_{\langle 3,3,2\rangle}\right\}.$$

S.Example 14. Using S.Example 8--13, we have

$$A_3^{con}=A_3^{con}\left(\mathbb{a}_{\langle 1,1\rangle}\right)\cup A_3^{con}\left(\mathbb{a}_{\langle 2,1\rangle}\right)\cup A_3^{con}\left(\mathbb{a}_{\langle 2,2\rangle}\right)\cup A_3^{con}\left(\mathbb{a}_{\langle 3,1\rangle}\right)\cup A_3^{con}\left(\mathbb{a}_{\langle 3,2\rangle}\right)\cup A_3^{con}\left(\mathbb{a}_{\langle 3,3\rangle}\right)$$
$$=\left\{\mathbb{a}_{\langle 1,1,1\rangle}\right\}\cup\left\{\mathbb{a}_{\langle 2,1,1\rangle}\right\}\cup\left\{\mathbb{a}_{\langle 2,2,1\rangle},\mathbb{a}_{\langle 2,2,2\rangle},\mathbb{a}_{\langle 2,2,3\rangle}\right\}\cup\left\{\mathbb{a}_{\langle 3,1,1\rangle}\right\}\cup\left\{\mathbb{a}_{\langle 3,2,1\rangle},\mathbb{a}_{\langle 3,2,2\rangle}\right\}\cup\left\{\mathbb{a}_{\langle 3,3,1\rangle},\mathbb{a}_{\langle 3,3,2\rangle}\right\}$$
$$=\left\{\begin{array}{l}\begin{bmatrix}10010000000,01000100000,10001010000,01100000000,10010010000,01000010000,\\10000000000,01100100000,10011010000,01000000000,10000010000,01100010000\end{bmatrix},\\ [\![00000100000,00001100000,00001000000]\!],\\ [\![00000010000]\!],[\![00000010011]\!],[\![00000010010,00000010001]\!],\\ [\![11001000000,11101000000,11111000000,11011000000]\!],\\ \begin{bmatrix}11000010000,11100000000,\\11110010000,11010000000\end{bmatrix},\begin{bmatrix}11000010010,11100000001,\\11110010010,11010000001\end{bmatrix}\\ \begin{bmatrix}11000000000,11100010000,\\11110000000,11010010000\end{bmatrix},\begin{bmatrix}11000000001,11100010010,\\11110000001,11010010010\end{bmatrix}\end{array}\right\}.$$

Therefore the original network V has 10 global attractors

$$a_{\langle 1,1,1\rangle},a_{\langle 2,1,1\rangle}\ a_{\langle 2,2,1\rangle},a_{\langle 2,2,2\rangle},a_{\langle 2,2,3\rangle},a_{\langle 3,1,1\rangle},a_{\langle 3,2,1\rangle},a_{\langle 3,2,2\rangle},a_{\langle 3,3,1\rangle},a_{\langle 3,3,2\rangle}$$

S.Remark 2. We summarize the symbols for local attractors used for concatenation to construct global attractors as in S.Table1. In particular, the last local attractors become the global attractors.

Net	Attractor									
$V_{1,1}$	$a_{\langle 1\rangle}$	$a_{\langle 2\rangle}$				$a_{\langle 3\rangle}$				
$V_{2,1}$	$a^1_{\langle 1:2,1\rangle}$	$a^1_{\langle 2:2,1\rangle}$				$a^1_{\langle 3:2,1\rangle}$				
$V_{2,2}$	$a^1_{\langle 1:2,2\rangle}$	$a^1_{\langle 2:2,2\rangle}$	$a^2_{\langle 2:2,2\rangle}$			$a^1_{\langle 3:2,2\rangle}$	$a^2_{\langle 3:2,2\rangle}$			
V_2	$a_{\langle 1,1\rangle}$	$a_{\langle 2,1\rangle}$	$a_{\langle 2,2\rangle}$			$a_{\langle 3,1\rangle}$	$a_{\langle 3,2\rangle}$		$a_{\langle 3,3\rangle}$	
$V_{3,1}$	$a^1_{\langle 1,1:3,1\rangle}$	$a^1_{\langle 2,1:3,1\rangle}$	$a^1_{\langle 2,2:3,1\rangle}$			$a^1_{\langle 3,1:3,1\rangle}$	$a^1_{\langle 3,2:3,1\rangle}$		$a^1_{\langle 3,3:3,1\rangle}$	
$V_{3,2}$	$a^1_{\langle 1,1:3,2\rangle}$	$a^1_{\langle 2,1:3,2\rangle}$	$a^1_{\langle 2,2:3,2\rangle}$	$a^2_{\langle 2,2:3,2\rangle}$	$a^3_{\langle 2,2:3,2\rangle}$	$a^1_{\langle 3,1:3,2\rangle}$	$a^1_{\langle 3,2:3,2\rangle}$	$a^2_{\langle 3,2:3,2\rangle}$	$a^1_{\langle 3,3:3,2\rangle}$	$a^2_{\langle 3,3:3,2\rangle}$
V_3	$a_{\langle 1,1,1\rangle}$	$a_{\langle 2,1,1\rangle}$	$a_{\langle 2,2,1\rangle}$	$a_{\langle 2,2,2\rangle}$	$a_{\langle 2,2,3\rangle}$	$a_{\langle 3,1,1\rangle}$	$a_{\langle 3,2,1\rangle}$	$a_{\langle 3,2,2\rangle}$	$a_{\langle 3,3,1\rangle}$	$a_{\langle 3,3,2\rangle}$

S.Table 1. Symbols for local attractors

S.Remark 3. We summarize the process of how we construct global attractors by concatenation of local attractors in S.Fig. 3.

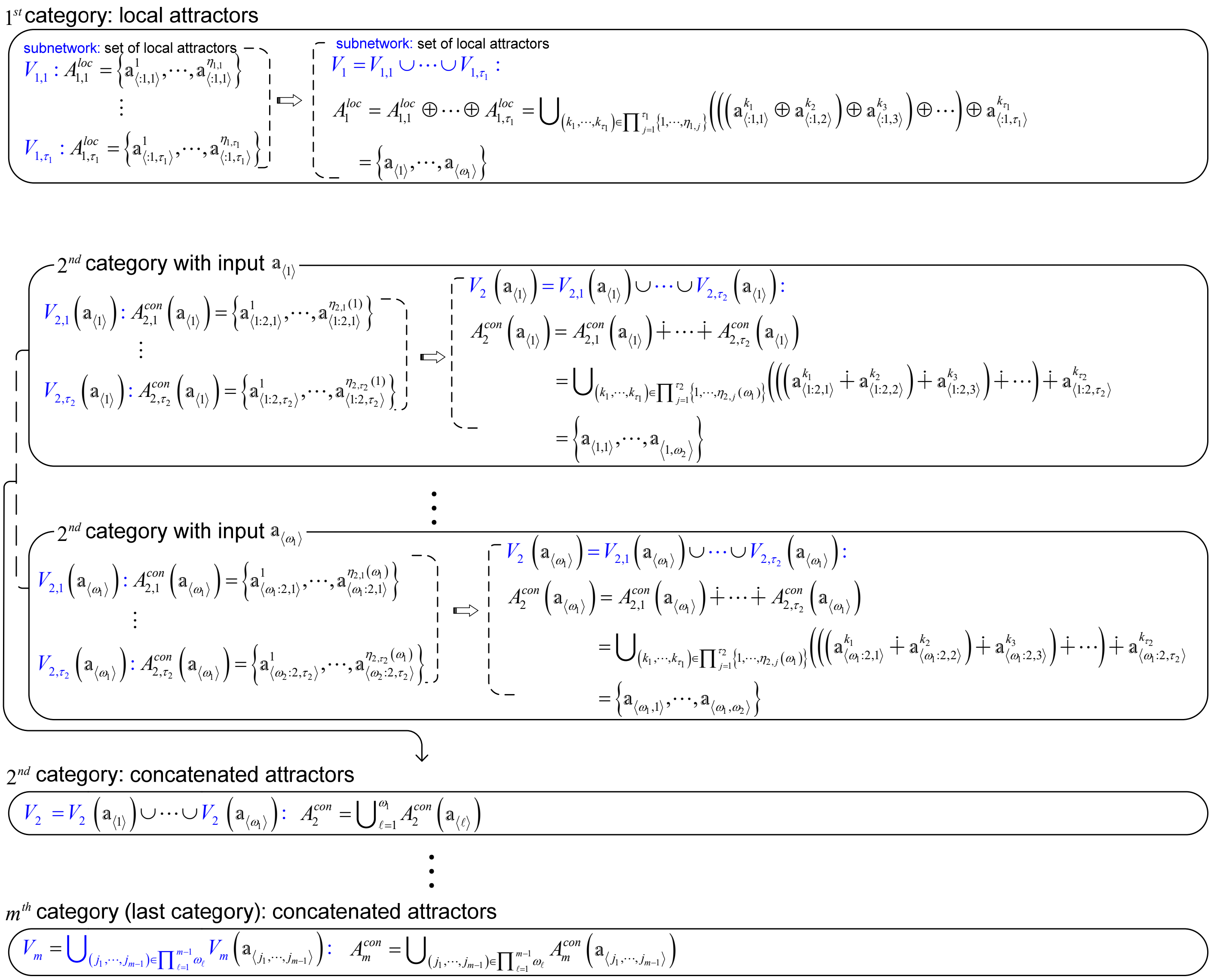

S.Fig 3. Summary of the process to concatenation local attractors

In the following we write a theorem that the last local attractors become the global attractors.

S.Theorem 2. Let $\bigcup_{i=1}^{m}\{V_{i,1},\cdots,V_{i,\rho_i}\}$ be a hierarchical partition of V, V_i the i-th category and A_i^{con} the set of concatenated attractors of V_i for $2 \le i \le m$.

(1) $A_{\lambda,\varsigma}^{con}\left(a_{\langle j_1,\cdots,j_{\lambda-1}\rangle}\right)$ is the set of attractors of $\left(\bigcup_{\substack{1\le i\le\lambda-1\\1\le k\le\delta_i}} V_{i,k}\left(a_{\langle j_1,\cdots,j_{\lambda-1}\rangle}\right)\right)\cup V_{\lambda,\varsigma}\left(a_{\langle j_1,\cdots,j_{\lambda-1}\rangle}\right)$.

(2) $A_{\lambda}^{con}\left(a_{\langle j_1,\cdots,j_{\lambda-1}\rangle}\right)$ is the set of attractors of $\left(\bigcup_{\substack{1\le i\le\lambda-1\\1\le k\le\delta_i}} V_{i,k}\left(a_{\langle j_1,\cdots,j_{\lambda-1}\rangle}\right)\right)\cup V_{\lambda}\left(a_{\langle j_1,\cdots,j_{\lambda-1}\rangle}\right)$.

(3) A_m^{con} is the set of attractors of V.

Proof. (1)

The set of input nodes to $\left(\bigcup_{\substack{1\le i\le\lambda-1\\1\le k\le\delta_i}} V_{i,k}\left(a_{\langle j_1,\cdots,j_{\lambda-1}\rangle}\right)\right)\cup V_{\lambda,\varsigma}\left(a_{\langle j_1,\cdots,j_{\lambda-1}\rangle}\right)$ is empty.

Letting $\left(\bigcup_{\substack{1\le i\le\lambda-1\\1\le k\le\delta_i}} V_{i,k}\left(\mathbb{a}_{\langle j_1,\cdots,j_{\lambda-1}\rangle}\right)\right)\cup V_{\lambda,\varsigma}\left(\mathbb{a}_{\langle j_1,\cdots,j_{\lambda-1}\rangle}\right)=\{y_1,\cdots,y_s\}$ for some s, we have the update rules for $\left(\bigcup_{\substack{1\le i\le\lambda-1\\1\le k\le\delta_i}} V_{i,k}\left(\mathbb{a}_{\langle j_1,\cdots,j_{\lambda-1}\rangle}\right)\right)\cup V_{\lambda,\varsigma}\left(\mathbb{a}_{\langle j_1,\cdots,j_{\lambda-1}\rangle}\right)$:

$$y_1^{t+1}=\tilde{f}_{i_1}\left(y_1^t,\cdots,y_s^t\right),\cdots,y_s^{t+1}=\tilde{f}_{i_s}\left(y_1^t,\cdots,y_s^t\right)$$

for some $\{i_1,\cdots,i_s\}\subset\{1,\cdots,n\}$.

Since the set $\left\{\tilde{f}_{i_1},\cdots,\tilde{f}_{i_s}\right\}$ is the union of the update rule functions for $V_{i,k}\left(\mathbb{a}_{\langle j_1,\cdots,j_{\lambda-1}\rangle}\right)$ and $V_{\lambda,\varsigma}\left(\mathbb{a}_{\langle j_1,\cdots,j_{\lambda-1}\rangle}\right)$ with $1\le i\le\lambda-1$ and $1\le k\le\delta,$ the set of concatenated attractors of $\left(\bigcup_{\substack{1\le i\le\lambda-1\\1\le k\le\delta_i}} V_{i,k}\left(\mathbb{a}_{\langle j_1,\cdots,j_{\lambda-1}\rangle}\right)\right)\cup V_{\lambda,\varsigma}\left(\mathbb{a}_{\langle j_1,\cdots,j_{\lambda-1}\rangle}\right)$ is $A_{\lambda,\varsigma}^{con}\left(\mathbb{a}_{\langle j_1,\cdots,j_{\lambda-1}\rangle}\right)$.

Similarly we can prove (2) and (3).

Q.E.D.

Finally we can describe the algorithm for construction of global attractors as follows.

Algorithm for finding attractors based on the hierarchical partition of the first type

Let $\bigcup_{i=1}^m\left\{V_{i,1},\cdots,V_{i,\rho_i}\right\}$ be a hierarchical partition of V.

Step1. Construction of attractors in the 1st-category

Step1-1. Find the set of local attractors of start subnetworks $V_{1,k}$ and denote it by

$$A_{1,k}^{loc}=\left\{\mathbb{a}_{\langle :1,k\rangle}^1,\cdots,\mathbb{a}_{\langle :1,k\rangle}^{\eta_{1,k}}\right\}.$$

Step1-2. Find the set of local attractors of the subnetwork $V_1=V_{1,1}\cup\cdots\cup V_{1,\tau_1}$, the union of all subnetworks in the 1st-category, and denote it by

$$A_1^{loc}=A_{1,1}^{loc}\oplus\cdots\oplus A_{1,\tau_1}^{loc}=\left\{\mathbb{a}_{\langle 1\rangle},\cdots,\mathbb{a}_{\langle\omega_1\rangle}\right\}$$

where the operator $\oplus$ is defined in S.Definition 6.

Step2. Construction of attractors in the i-th category for $2\le i\le m$

Step2-1. Find the three sets of concatenated, partial and local attractors of start subnetworks $V_i\left(\mathbb{a}_{\langle j_1,\cdots,j_{i-1}\rangle}\right)$ and denote them by $A_{i,k}^{con}\left(\mathbb{a}_{\langle j_1,\cdots,j_{i-1}\rangle}\right), A_{i,k}^{par}\left(\mathbb{a}_{\langle j_1,\cdots,j_{i-1}\rangle}\right)$ and $A_{i,k}^{loc}\left(\mathbb{a}_{\langle j_1,\cdots,j_{i-1}\rangle}\right)$, respectively, where

$$A_{i,k}^{con}\left(\mathbb{a}_{\langle j_1,\cdots,j_{i-1}\rangle}\right)=\left\{\mathbb{a}_{\langle j_1,\cdots,j_{i-1}:i,k\rangle}^1,\cdots,\mathbb{a}_{\langle j_1,\cdots,j_{i-1}:i,k\rangle}^{\eta_{i,k}(j_1,\cdots,j_{i-1})}\right\},\ 1\le k\le\omega_i.$$

Step2-2. Find the set of local attractors of the subnetwork

$$V_i\left(\mathbb{a}_{\langle j_1,\cdots,j_{i-1}\rangle}\right)=V_{i,1}\left(\mathbb{a}_{\langle j_1,\cdots,j_{i-1}\rangle}\right)\cup\cdots\cup V_{i,\tau_i}\left(\mathbb{a}_{\langle j_1,\cdots,j_{i-1}\rangle}\right),$$

which is the union of all subnetworks in the i-th category. Denote the set by

$$A_i^{con}\left(\mathbb{a}_{\langle j_1,\cdots,j_{i-1}\rangle}\right) = A_{i,1}^{con}\left(\mathbb{a}_{\langle j_1,\cdots,j_{i-1}\rangle}\right) \dot{+} \cdots \dot{+} A_{i,\tau_i}^{con}\left(\mathbb{a}_{\langle j_1,\cdots,j_{i-1}\rangle}\right)$$

where the operator $\dot{+}$ is defined in S.Definition 6.

Step2-3. Find the set of local attractors of the subnetwork

$$V_i = \bigcup_{(j_1,\cdots,j_{i-1})\in\prod_{\ell=1}^{i-1}\omega_\ell} V_i\left(\mathbb{a}_{\langle j_1,\cdots,j_{i-1}\rangle}\right)$$

and denote it by

$$A_i^{con} = \bigcup_{(j_1,\cdots,j_{i-1})\in\prod_{\ell=1}^{i-1}\omega_\ell} A_i^{con}\left(\mathbb{a}_{\langle j_1,\cdots,j_{i-1}\rangle}\right).$$

S. Remark 4. In order to find the attractor $\mathbb{a}_{\langle 3,2,1\rangle}$, we first choose the third attractor $\mathbb{a}_{\langle 3\rangle}$ in the 1st-category and use $\mathbb{a}_{\langle 3\rangle}$ as the input signal to the 2nd-category for finding the concatenated attractors $\mathbb{a}_{\langle 3,k\rangle}$ in the 2nd-category. Next, we choose the second concatenated attractor $\mathbb{a}_{\langle 3,2\rangle}$, which is used as the input signal to the 3rd-category for finding the concatenated attractors $\mathbb{a}_{\langle 3,2,1\rangle}$ in the 3rd-category. Therefore two attractors $\mathbb{a}_{\langle 2,2,1\rangle}$ and $\mathbb{a}_{\langle 3,2,1\rangle}$ can be founded hierarchically and independently from the first category, so that it is possible to use parallel computation for finding global attractors in our framework. In the case of $\mathbb{a}_{\langle 3,2,1\rangle}$ and $\mathbb{a}_{\langle 3,1,1\rangle}$, they can be founded hierarchically and independently from the 2nd-category.
