# Supplementary material for: An efficient algorithm for identifying primary phenotype attractors of a large-scale Boolean network
Source: BMC Syst Biol. 2016 Oct 7;10:95. doi: 10.1186/s12918-016-0338-4 (PMC5055661; doi:10.1186/s12918-016-0338-4)
Supplement: Additional file 9: — Attractors for apoptosis in the MAPK network. (PDF 189 kb) [file 12918_2016_338_MOESM9_ESM.pdf]

An Efficient Algorithm for Identifying Primary Phenotype Attractors of a Large-Scale Boolean Network

Sang-Mok Choo[1] and Kwang-Hyun Cho[2,*]

[1]Department of Mathematics, University of Ulsan, Ulsan 44610, Republic of Korea

[2]Department of Bio and Brain Engineering, Korea Advanced Institute of Science and Technology (KAIST), Daejeon 34141, Republic of Korea

Supporting information

Apoptosis attractors of the MAPK network

* Corresponding author, E-mail: ckh@kaist.ac.kr, Phone: +82-42-350-4325, Fax: +82-42-350-4310, Web: http://sbie.kaist.ac.kr

To find attractors for apoptosis phenotype we use the simulation condition r4 in S3 Dataset of [2], which is FGFR3 perturbation with setting values of the four stimuli to zero. Inserting the values (FGFR3, TGFBR stimulus, EGFR stimulus, FGFR3 stimulus, DNA damage)=(1,0,0,0,0) of the external nodes into the update rules for the original network in Additional file 3(a), we found the external condition : the external, secondary-external nodes (ESENs) and the semi-simplified update rules for nodes except ESENs in Additional file 3(b). Due to the update rule Apoptosis*=!BCL2 & !ERK & FOXO3 & p53, inserting the values (BCL2, ERK, FOXO3, p53)=(0,0,1,1) into the semi-simplified update rules, we found the phenotype condition: the phenotype, secondary-phenotype nodes (PSPNs) and the fully-simplified update rules for 6 nodes in Fig. 6 with no secondary-phenotype equations in Additional file 3(c). The fully-simplified update rules yield the HPFP for apoptosis in Fig. 6, where the HPFP has 4 categories with the maximum value 2 of the numbers of nodes in the SCCs.

The SCCs with more than one node in the HPFP are $V_{1,1}$ ={ FRS2, GRB2} and $V_{3,1}$ = {PI3K, GAB1}. The fully-simplified update rules for (FRS2, GRB2) yield that $V_{1,1}$ has only one attractor $[\![00,\ 10,\ 11,\ 01]\!]$, which is cyclic with period 4 in Additional file 3(d). The signal from {GRB2} in $V_{1,1}$ is transmitted to $V_{2,1} = \{\mathrm{SOS}\}$ with SOS*=GRB2. Then $V_{2,1}$ has the unique attractor $[\![1,0,0,1]\!]$. The input signals from {SOS, GRB2} yield a unique attractor of $V_{3,1}$, which is the point attractor $[\![11]\!]$ for (PI3K, GAB1) in Additional file 3(e). Since there is no secondary-phenotype equation, we do not check whether secondary-phenotype equations are satisfied. Therefore we can construct a unique global attractor for apoptosis, which is cyclic with period 4 in Additional file 3(f). We confirm the concatenated state vector becomes a global attractor by applying the original update rules in Additional file 3(f).
