# Supplementary material for: An efficient algorithm for identifying primary phenotype attractors of a large-scale Boolean network
Source: BMC Syst Biol. 2016 Oct 7;10:95. doi: 10.1186/s12918-016-0338-4 (PMC5055661; doi:10.1186/s12918-016-0338-4)
Supplement: Additional file 10: — Attractors for apoptosis in the CACC network. (PDF 193 kb) [file 12918_2016_338_MOESM10_ESM.pdf]

An Efficient Algorithm for Identifying Primary Phenotype Attractors of a Large-Scale Boolean Network

Sang-Mok Choo[1] and Kwang-Hyun Cho[2,*]

[1]Department of Mathematics, University of Ulsan, Ulsan 44610, Republic of Korea

[2]Department of Bio and Brain Engineering, Korea Advanced Institute of Science and Technology (KAIST), Daejeon 34141, Republic of Korea

Supporting information

Apoptosis attractors of the CACC network

[*] Corresponding author, E-mail: ckh@kaist.ac.kr, Phone: +82-42-350-4325, Fax: +82-42-350-4310, Web: http://sbie.kaist.ac.kr

Under the strongest tumour-promoting microenvironment (fixing DC at ON) for premalignant intestinal epithelial cells (fixing APC at ON) as in [3], our method is applied to find attractors for proliferation with the update rules in Additional file 5(a). Inserting the values (DC, APC)=(1,1) into the original update rules in Additional file 5(a), we found the external condition: the external, secondary-external nodes (ESENs) and the semi-simplified update rules for nodes except ESENs in Additional file 5(b). Due to Apoptosis*=CASP3, inserting the value CASP3=1 into the semi-simplified update rules, we found the phenotype condition : the phenotype, secondary-phenotype nodes (PSPNs) and the fully-simplified update rules for 42 nodes in Fig. 9 with the two secondary-phenotype equations

0=(NFKB or STAT3) and not SMAC,

1= CASP8 or CASP9

in Additional file 5(c). The fully-simplified update rules yield the HPFP for apoptosis in Fig. 9, where the HPFP has 15 categories with the maximum value 7 of the numbers of nodes in the SCCs. The yellow boxes on the five nodes STAT3, NFKB, SMAC, CASP8 and CASP9 in Fig. 9 denote the nodes contained in the secondary-phenotype equation.

The SCCs with more than one node in the HPFP are

$$V_{1,1}=\{\text{IFNG,CTL}\},\ V_{1,2}=\{\text{SOCS,STAT3,JAK}\},$$
$$V_{4,1}=\{\text{IKB,IKK,NFKB,TNFR,TNFA,MAC,CCL2}\},$$
$$V_{10,1}=\{\text{P53,PTEN}\}.$$

The fully-simplified update rules for (CTL, IFNG) is CTL*=IFNG and IFNG*=CT, so that $V_{1,1}$ has two point attractors and one cyclic attractor of length 2

$$a_1=[\![00]\!],a_2=[\![11]\!],a_3=[\![01,10]\!]$$

in Additional file 5(d). The fully-simplified update rules for (SOCS,STAT3,JAK) yields that $V_{1,2}$ has two cyclic attractors

$$b_1=[\![010,\ 101]\!],\ b_2=[\![111,\ 110,\ 100,\ 000,\ 001,\ 011]\!]$$

in Additional file 5(e). Hence we can construct attractors for (CTL, IFNG, SOCS, STAT3, JAK) in $V_{1,1}\cup V_{2,1}$

$$\{a_1,a_2,a_3\}\oplus\{b_1,b_2\}=\{a_i\oplus b_j \mid 1\le i\le 3,1\le j\le 2\}.$$

Here $a_3\oplus b_1$ has the two cyclic attractors of length 2 and $a_3\oplus b_2$ has the two cyclic attractors of length 6, so that $V_{1,1}\cup V_{1,2}$ has the eight attractors for apoptosis.

Starting from each of the eight local attractors and concatenating local attractors of other subnetworks in the HPFP in Additional files 5(f)--5(m), we found that all the concatenated attractors do not satisfy the two secondary-phenotype equations in Additional files 5(f)--5(m). Therefore there exists no global attractor for apoptosis, which was checked by using the random sampling method.
