# Supplementary material for: An efficient algorithm for identifying primary phenotype attractors of a large-scale Boolean network
Source: BMC Syst Biol. 2016 Oct 7;10:95. doi: 10.1186/s12918-016-0338-4 (PMC5055661; doi:10.1186/s12918-016-0338-4)
Supplement: Additional file 12: — Validation of the proposed algorithm for two small and medium networks. (PDF 158 kb) [file 12918_2016_338_MOESM12_ESM.pdf]

An Efficient Algorithm for Identifying Primary Phenotype Attractors of a Large-Scale Boolean Network

Sang-Mok Choo[1] and Kwang-Hyun Cho[2,*]

[1]Department of Mathematics, University of Ulsan, Ulsan 44610, Republic of Korea

[2]Department of Bio and Brain Engineering, Korea Advanced Institute of Science and Technology (KAIST), Daejeon 34141, Republic of Korea

Supporting information

Validation of the proposed algorithm

[*] Corresponding author, E-mail: ckh@kaist.ac.kr, Phone: +82-42-350-4325, Fax: +82-42-350-4310, Web: http://sbie.kaist.ac.kr

For the network in Fig. 3(a) with 11 nodes and the update rules in Additional file 7, we found 10 attractors in Additional file 8 as follows:

$$\left(\mathrm{a}_{\langle 1,1,1\rangle},\mathrm{a}_{\langle 2,1,1\rangle},\mathrm{a}_{\langle 2,2,1\rangle},\mathrm{a}_{\langle 2,2,2\rangle},\mathrm{a}_{\langle 2,2,3\rangle},\mathrm{a}_{\langle 3,1,1\rangle},\mathrm{a}_{\langle 3,2,1\rangle},\mathrm{a}_{\langle 3,2,2\rangle},\mathrm{a}_{\langle 3,3,1\rangle},\mathrm{a}_{\langle 3,3,2\rangle}\right)$$
$$=\left(\begin{array}{l}
\left[\!\left[\begin{array}{l}10010000000,01000100000,10001010000,01100000000,10010010000,01000010000,\\ 10000000000,01100100000,10011010000,01000000000,10000010000,01100010000\end{array}\right]\!\right],\\
[\![00000100000,00001100000,00001000000]\!],\\
[\![00000010000]\!],[\![00000010011]\!],[\![00000010010,00000010001]\!],\\
[\![11001000000,11101000000,11111000000,11011000000]\!],\\
\left[\!\left[\begin{array}{l}11000010000,11100000000,\\ 11110010000,11010000000\end{array}\right]\!\right],\left[\!\left[\begin{array}{l}11000010010,11100000001,\\ 11110010010,11010000001\end{array}\right]\!\right]\\
\left[\!\left[\begin{array}{l}11000000000,11100010000,\\ 11110000000,11010010000\end{array}\right]\!\right],\left[\!\left[\begin{array}{l}11000000001,11100010010,\\ 11110000001,11010010010\end{array}\right]\!\right]
\end{array}\right)$$

Here, we used the notation $\mathrm{a}_{\langle 3,3,2\rangle}=\left[\!\left[\begin{array}{l}11000000001,11100010010,\\ 11110000001,11010010010\end{array}\right]\!\right]$ for the cyclic attractor with length 4 instead of representing it as $[\![11000000001,11100010010,11110000001,11010010010]\!]$.

On the other hand, we obtained the following attractors by applying a full search algorithm to the network:

attractor1	16										$\mathrm{a}_{\langle 2,2,1\rangle}$
attractor2	19										$\mathrm{a}_{\langle 2,2,2\rangle}$
attractor3	64	32	96								$\mathrm{a}_{\langle 2,1,1\rangle}$
attractor4	18	17									$\mathrm{a}_{\langle 2,2,3\rangle}$
attractor5	1232	512	1040	784	1152	544	1104	768	1168	528	$\mathrm{a}_{\langle 1,1,1\rangle}$
attractor6	1680	1536	1808	1920							$\mathrm{a}_{\langle 3,3,1\rangle}$
attractor7	1682	1537	1810	1921							$\mathrm{a}_{\langle 3,3,2\rangle}$
attractor8	1664	1552	1792	1936							$\mathrm{a}_{\langle 3,2,1\rangle}$
attractor9	1665	1554	1793	1938							$\mathrm{a}_{\langle 3,2,2\rangle}$
attractor10	1728	1600	1856	1984							$\mathrm{a}_{\langle 3,1,1\rangle}$

In the above, we used decimal numbers to represent the 11 bits binary numbers. For instance, in attractor 1, 16 denotes 00000010000 which means the attractor $\mathrm{a}_{\langle 2,2,1\rangle}$. From these, we can confirm that the attractors obtained by the full search algorithm are the same as the attractors obtained by applying our algorithm.

The above network has no output nodes, so it is appropriate for the validation of concatenating local attractors obtained from the HPFP. For the validation of PSPNs as well as the concatenation, we have employed another network from [10] that has an output node representing cell proliferation, as shown below.

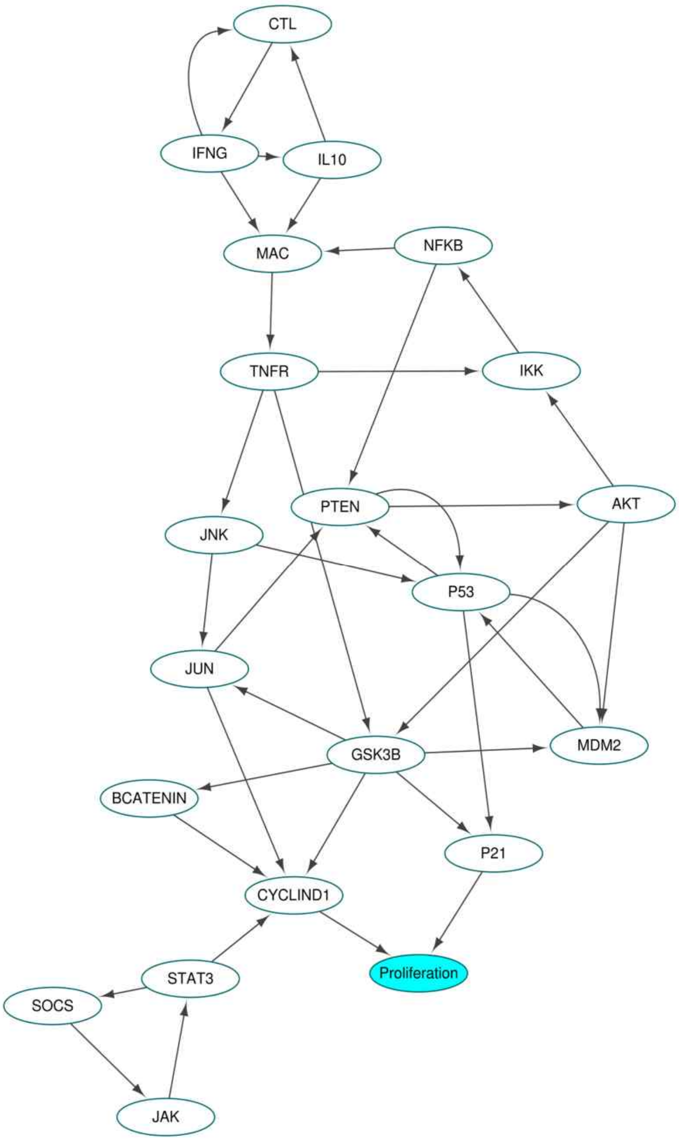

The authors of [10] presented all attractors for proliferation, which are two cyclic attractors with length 2 and 6, respectively (see Figure 3 (c) and Supplementary Table S6 in [10]). To find out all attractors for proliferation in this network model, we applied our method to this network and found the following two cyclic attractors with the lengths of 2 and 6

	AKT	BCATENIN	CTL	CYCLIND1	GSK3B	IFNG	IKK	IL10	**JAK**	JNK	JUN	MAC	MDM2	NFKB	P21	P53	PTEN	**SOCS**	**STAT3**	TNFR	PROLIFERAT
t=1	1	1	0	1	0	0	1	1	**0**	0	0	0	0	1	0	0	0	**0**	**1**	0	1
t=2	1	1	0	1	0	0	1	1	**1**	0	0	0	0	1	0	0	0	**1**	**0**	0	1

and

	AKT	BCATENIN	CTL	CYCLIND1	GSK3B	IFNG	IKK	IL10	**JAK**	JNK	JUN	MAC	MDM2	NFKB	P21	P53	PTEN	**SOCS**	**STAT3**	TNFR	PROLIFER
t=1	1	1	0	1	0	0	1	1	**1**	0	0	0	0	1	0	0	0	**1**	**1**	0	1
t=2	1	1	0	1	0	0	1	1	**0**	0	0	0	0	1	0	0	0	**1**	**1**	0	1
t=3	1	1	0	1	0	0	1	1	**0**	0	0	0	0	1	0	0	0	**1**	**0**	0	1
t=4	1	1	0	1	0	0	1	1	**0**	0	0	0	0	1	0	0	0	**0**	**0**	0	1
t=5	1	1	0	1	0	0	1	1	**1**	0	0	0	0	1	0	0	0	**0**	**0**	0	1
t=6	1	1	0	1	0	0	1	1	**1**	0	0	0	0	1	0	0	0	**0**	**1**	0	1

We have described in detail the process of finding out the attractors in Additional file 10.

By comparing the above two cyclic attractors with the two cyclic attractors for proliferation shown in Figure 3(c) and Supplementary Table S6 (as presented in [10]), we can confirm that the set of attractors for proliferation obtained by applying our method is the same as the known attractors representing proliferation in [10].
